# Combining magnetic resonance imaging and evoked potentials enhances machine learning prediction of multiple sclerosis disability worsening

**DOI:** 10.3389/fimmu.2026.1625837

**Published:** 2026-03-11

**Authors:** Sofie Aerts, Lorin Werthen-Brabants, Hamza Khan, Diana L. Giraldo, Edward De Brouwer, Lotte Geys, Veronica Popescu, Jan Sijbers, Henry C. Woodruff, Tom Dhaene, Dirk Deschrijver, Bart Van Wijmeersch, Philippe Lambin, Liesbet M. Peeters

**Affiliations:** 1 University MS Centre (UMSC), Hasselt-Pelt, Belgium; 2 UHasselt, Biomedical Research Institute (BIOMED), Diepenbeek, Belgium; 3 Noorderhart, Rehabilitation and MS Centre, Pelt, Belgium; 4 UHasselt, Rehabilitation Research Centre (REVAL), Faculty of Rehabilitation Sciences, Diepenbeek, Belgium; 5 IDLab, Ghent University - Imec, Ghent, Belgium; 6 UHasselt, Data Science Institute (DSI), Diepenbeek, Belgium; 7 The D-Lab, Department of Precision Medicine, GROW – Research Institute for Oncology & Reproduction, Maastricht University, Maastricht, Netherlands; 8 Imec-Vision Lab, Universiteit Antwerpen, Antwerp, Belgium; 9 µNEURO Research Centre of Excellence, University of Antwerp, Antwerp, Belgium; 10 ESAT-STADIUS, KU Leuven, Leuven, Belgium; 11 Department of Radiology and Nuclear Imaging, GROW – Research Institute for Oncology & Reproduction, Maastricht University Medical Centre, Maastricht, Netherlands

**Keywords:** disease worsening, evoked potentials, machine learning, magnetic resonance imaging, multiple sclerosis, prognosis, radiomics

## Abstract

**Introduction:**

Predicting long-term disability progression in multiple sclerosis (MS) remains a significant challenge. Existing prognostic models often rely on single-modality data or conventional measures, such as lesion count on magnetic resonance imaging (MRI) or latency values from evoked potentials (EPs), overlooking subclinical disease progression. This study aimed to develop a multimodal machine learning (ML) pipeline integrating clinical, high-dimensional MRI, and motor EP time-series (EPTS) features to predict disability worsening in MS.

**Methods:**

A retrospective cohort of 127 people with MS (PwMS; 424 episodes) from a tertiary MS center in Belgium was used, including clinical data, T2-weighted fluid-attenuated inversion recovery MRI, and motor EPs. Disability worsening was defined as a change in the expanded disability status scale (EDSS) over two years, stratified by baseline EDSS. MRI features included 42 anatomical and lesion volumes and 100 radiomic descriptors from lesions and the normal-appearing white matter (NAWM). EPTS features included latency, peak-to-peak amplitude (PPA), and high-dimensional descriptors selected using highly comparative time-series analysis (HCTSA) and Boruta. ML models (Light Gradient Boosting Machine (LGBM), random forest, logistic regression) were trained using 20×repeated stratified 3-fold cross-validation. Performance was evaluated using the area under the receiver operating characteristic curve (AUROC), average precision (AP), and Brier score. SHapley Additive exPlanations (SHAP) were used for interpretability.

**Results:**

Across 96 model configurations, models combining MRI and EPTS features, with or without clinical data, consistently outperformed single-modality models across AUROC, AP, and Brier score, regardless of algorithm or feature representation. The best-performing model (Brier score = 0.062) was an LGBM using combined MRI and EPTS data. MRI radiomics dominated feature importance, especially shape- and texture-based features from NAWM and lesion regions. EPTS features, particularly waveform dynamics (e.g., Sliding Window) and PPA, provided complementary value and improved sensitivity. EPTS-only models showed the highest AUROC, but combined models achieved the best overall balance across all performance metrics.

**Conclusion:**

This is the first study to integrate clinical, MRI radiomics, and motor EPTS features in an ML pipeline for MS prognosis. Combining structural and functional subclinical markers improves the prediction of disability worsening and supports multimodal monitoring for personalized care.

## 1 Introduction

Multiple sclerosis (MS) is a chronic, immune-mediated condition of the central nervous system (CNS) that leads to progressive neurological disability through inflammation, demyelination, and neurodegeneration (1). As of 2020, the global prevalence of multiple sclerosis exceeded 2.8 million individuals, with numbers continuing to rise (2). CNS lesions disrupt neural transmission, producing heterogeneous clinical manifestations that reflect the extent and distribution of CNS involvement (3). Traditionally, MS has been divided into relapsing and progressive forms, but growing evidence suggests a continuous disease spectrum driven by overlapping pathological and reparative processes (4, 5). While no definitive cure exists, disease-modifying therapies (DMTs) can reduce disease activity and delay disability progression, particularly in relapsing MS, though effective options for progressive MS remain limited (6).

To date, predicting the MS disease course with sufficient accuracy remains impossible in clinical practice. This uncertainty exacerbates psychological distress and adversely affects the quality of life for people with MS (PwMS) (7, 8). The heterogeneity of MS also challenges clinicians attempting to personalize care. Consequently, there is a pressing need for robust prognostic prediction models that can identify PwMS at higher risk of rapid disease worsening and inform prognosis-driven treatment (9). Indeed, the timely initiation of high-efficacy DMTs within an optimal therapeutic window is paramount for PwMS with poor prognostic markers, as it can maximize long-term benefits and prevent irreversible disability progression (10, 11).

In light of these challenges, machine learning (ML) represents a powerful tool for enhancing the prediction of the MS disease course (12–14). Yet, wide variability in both predictors and outcomes, coupled with a high overall risk of bias and limited external validation, continues to hamper the clinical utility of current prediction models (15). In addition, most predictive models for MS to date have relied on a single data modality, often restricted to clinical or magnetic resonance imaging (MRI) biomarkers, or *features*. This approach may be insufficient given the increasing recognition of *smoldering-associated worsening*, characterized by subclinical pathological activity that persists independently of overt relapses and drives gradual functional decline (4, 16). Since smoldering MS is not always captured through standard clinical evaluations or conventional MRI, more holistic models are required.

To address the limited ability of existing predictive models to detect smoldering MS, this study proposes a multimodal ML pipeline for predicting MS disability worsening, measured by changes in the expanded disability status scale (EDSS) (17). Specifically, we integrate clinical data, MRI features, and evoked potential (EP) features, thereby providing a more complete picture of the multifaceted pathophysiology of MS progression. The rationale for integrating these modalities, along with their relevance to disease monitoring and prognosis, is detailed in the following paragraphs.

In prior work, De Brouwer et al. demonstrated the feasibility of using ML to predict disability worsening, as measured by the EDSS, over a two-year period. Utilizing a static clinical feature set, including age, gender and Kurtzke functional system scores, from 15,240 PwMS in the MSBase Registry (18), their best-performing model, a Bayesian neural network (19), achieved an area under the receiver operating characteristic curve (AUROC) of 0.68 (± 0.02 standard deviation (SD)) and an area under the precision- recall curve (AUPRC) of 0.23 (± 0.02 SD) (20). Our study expands upon this important benchmark by incorporating MRI and electrophysiological features alongside clinical features.

MRI remains central to MS diagnosis and monitoring (21). However, conventional MRI measures, such as lesion count or volume, fail to capture diffuse neurodegenerative changes, contributing to the well-known *clinico-radiological paradox* (22, 23). *Radiomics* can complement these conventional measures by extracting a large number of features from medical images, providing deeper insights into demyelination and neurodegeneration in MS. Khan et al. demonstrated that radiomics features from T2-weighted fluid-attenuated inversion recovery (FLAIR) MRI improved disability worsening prediction in MS over clinical features alone (24). Their best model, a Light Gradient Boosting Machine (LGBM (25)) trained on radiomics and clinical features, achieved an AUROC of 0.64 (± 0.02 SD) and AUPRC of 0.20 (± 0.3 SD). Texture-based radiomics features from white matter lesions (WML) and normal-appearing white matter (NAWM) were key predictors. However, MRI-based models alone may not sufficiently detect early functional changes. Integrating additional modalities is therefore essential to move beyond a purely clinical-radiological understanding of MS (26, 27).

EPs are electrophysiological measurements that assess nerve signal conduction by applying sensory or motor stimuli and recording the resulting electrical activity along CNS pathways. The resulting waveform, or *EP time series (EPTS)*, captures changes in electrical potential after stimulation (28). While EPs have long been used in MS monitoring, their prognostic value remains debated (29–31). Nevertheless, they provide quantitative measures of CNS integrity and can detect damage before clinical symptoms manifest (32). Among various EP types, motor EPs (MEPs) are particularly relevant to MS-related disability. MEPs, recorded via transcranial magnetic stimulation (TMS) applied to the motor cortex, assess corticospinal tract integrity, with alterations linked to motor function decline (33, 34). Conventional EP analysis relies on simplified measures such as latency, peak-to-peak amplitude (PPA), and presence of dispersion, which may not fully capture the complexity of the raw EPTS (35). Yperman et al. introduced an ML framework that analyzed the entire EPTS rather than relying solely on simplified measures, achieving notable improvements in EDSS worsening prediction (Random Forest (RF) classifier (36), AUROC = 0.75 ± 0.07 SD) (37). Their findings demonstrate the potential of high-dimensional EPTS analysis to detect neurophysiological changes that precede clinical deterioration in MS.

While the individual contributions of clinical, MRI, and EP features to predicting MS disability worsening have been demonstrated, each modality alone provides only a partial view of disease progression. In clinical practice, clinicians integrate multiple data sources, including medical history, imaging, electrophysiology, and laboratory findings, to construct a comprehensive assessment of patient disease status and inform treatment decisions. Multimodal ML aims to replicate this integrative approach by combining heterogeneous data sources to improve predictive accuracy (38). Despite its increasing adoption in various medical fields, such as oncology and cardiology (38–40), multimodal ML remains relatively under-explored in MS research. Notably, a study involving 300 MS patients reported a 19% increase in AUROC when integrating structured electronic health records, imaging data, and clinical notes to predict EDSS milestones using deep learning (41). Another multicentric study of 322 MS patients, validated in an independent cohort of 271, developed ML models integrating clinical, imaging, and omics data, where the addition of omics data provided slight performance improvements in several model configurations (42). Likewise, a longitudinal study of 111 MS patients found that combining MRI, optical coherence tomography (OCT), and serum biomarkers significantly outperformed single-biomarker models in identifying individuals at risk for EDSS progression (43). Thus, the potential for multimodal ML in advancing MS care and prognostication is evident.

Hence, this study introduces a multimodal ML pipeline for predicting MS disability worsening on the EDSS by combining clinical, MRI radiomics, and motor EPTS features. To our knowledge, this represents the most comprehensive effort of this kind to date, offering three key contributions:

- We developed a multimodal ML pipeline that, to our knowledge, is the first to combine clinical, MRI radiomics, and motor EPTS data for predicting long-term disability worsening in MS. This approach captures both structural (MRI) and functional (EP) components of MS disease progression.
- We demonstrate that combining MRI radiomics and motor EPTS data, with or without adding clinical variables, consistently outperforms single-modality approaches across multiple model configurations, underscoring the complementary value of integrating diverse data modalities.
- We utilize the largest available longitudinal dataset to date that combines clinical, MRI radiomics, and motor EPTS data.

By addressing the limitations of single-modality predictions and incorporating both structural (MRI) and functional (EP) information, this study aims to advance prognostic modelling in MS and facilitate more personalized treatment strategies. The additional functional layer provided by motor EPTS may reveal early neurophysiological changes that precede overt clinical or radiological manifestations. Ultimately, this multimodal understanding of MS could enhance patient risk stratification, guide earlier treatment decisions, and eventually improve long-term outcomes.

## 2 Materials and methods

### 2.1 Study design and ethical considerations

This single-center observational study integrated three data modalities, more specifically clinical, MRI, and motor EP data, collected retrospectively at Noorderhart, Rehabilitation and MS Centre in Pelt, Belgium. To construct a combined dataset, all visits from the separate modalities were matched for each patient using unique patient identifiers. The final dataset included data collected during routine clinical care from June 2011 until May 2017. Clinical data were extracted from the MS data entry portal iMed (iMed, ^©^ 2022 MSBase Foundation, Australia) and included the following features: age, gender, and MS course. We adhered to the Transparent Reporting of a multivariable prediction model for Individual Prognosis Or Diagnosis (TRIPOD) (44) guidelines for reporting our study (see **Appendix 10**). This study has been approved by the Medical Ethics Committee of Hasselt University (CME2019/046). Due to the retrospective study design and the pseudonymization of data, obtaining patient informed consent was not required. All our preprocessing and model code are available at https://github.com/UHasselt-BiomedicalDataSciences/Radiomics_Epomics.

### 2.2 MRI data

Pseudonymized T2-weighted FLAIR MRI scans were acquired using a Philips Achieva 1.5T scanner with three distinct protocols (Protocols A, B, and C), depending on the acquisition date. Further details regarding the acquisition protocols are summarized in **Appendix 1**. A standardized pre-processing pipeline was established to prepare the MRI data for downstream analysis. First, all MRI images were denoised using adaptive non-local means filtering and corrected for bias field inhomogeneities with the N4 algorithm. Given that Protocols A and B consist of multiple low-resolution FLAIR images per session, a super resolution reconstruction (SRR) approach called Perceptual Super-Resolution in Multiple Sclerosis (45) was applied to enhance the through-plane resolution of multi-slice structural MRIs containing MS lesions. This technique harmonizes the spatial resolution across scans, which is crucial for downstream radiomics analysis and segmentation tasks (45).

To achieve whole-brain segmentation, we applied Sequence Adaptive Multimodal SEGmentation (SAM-SEG) to all FLAIR protocols (46). SAMSEG segmented 41 anatomical brain structures (see **Appendix 6**) and is adaptive to different MRI contrasts and scanner types. Among the segmented structures were normal-appearing white matter (NAWM), grey matter (GM), thalamus, and cerebrospinal fluid (CSF). Additionally, white matter lesions (WML) were segmented using the lesion prediction algorithm (47) as implemented in the lesion segmentation tool (LST) toolbox^1^, version 1.2.3 for SPM8^2^. Beyond segmentation, anatomical features and estimated lesion volumes obtained from SAMSEG and LST were normalized by the intracranial volume for comparability. Furthermore, intensity normalization was performed using adaptive histogram matching on all skull-stripped FLAIR images, as previously described by Khan et al. (24).

Adding to the anatomical features and lesion volumes, radiomics features were extracted using PyRa-diomics 2.20 from multiple brain structures, including WML, NAWM, GM, CSF, and thalamus. The extracted radiomics features belonged to six feature classes: shape-based features (48), first-order statistics (FO), grey level co-occurrence matrix (GLCM) (49), grey level run length matrix (GLRLM) (50), grey level size zone matrix (GLSZM) (51), and grey level dependence matrix (GLDM) (52). Shape-based features capture the geometric properties of a region of interest (ROI), FO detail the distribution of intensities, while the remaining four feature types (GLCM, GLRLM, GLSZM, and GLDM) are collectively referred to as textural features. Grey level features were computed by discretizing the images into 50 intensity bins, following the recommendations of the Image Biomarker Standardization Initiative (53). A total of 40 anatomical features, including lesion volume, along with 14 shape-based, 18 FO, and 68 textural features per ROI, were used as input to our ML pipeline. The details of the number of radiomics features extracted per class are provided in **Appendix 2**.

### 2.3 Motor EP data

We used motor EP data of which an anonymized version was previously published by Yperman et al. (28). In this dataset, MEPs were recorded bilaterally from the abductor pollicis brevis (APB) and abductor hallucis (AH) muscles following TMS of the corresponding motor cortex areas using two acquisition systems (devices A and B). Stimulation was delivered using a Magstim 2002 or Bistim device (The Magstim Company Ltd., Whitland, UK) with a 9 cm round coil at maximal stimulator output (2.2 T). Signals were recorded for 100 ms post-stimulation and digitized at either 20 kHz (device A) or 19.2 kHz (device B), with all 20 kHz recordings down-sampled to 19.2 kHz for consistency. Device A applied a 0.6 Hz – 10 kHz band-pass filter, whereas Device B employed a 100 Hz high-pass filter for noise reduction.

Electromyographic activity was recorded using three surface electrodes per limb: for upper limbs, electrodes were placed on the APB muscle, the proximal phalanx of the thumb, and the dorsum of the hand (ground); for lower limbs, on the AH muscle, the big toe, and the dorsum of the foot (ground). Stimulation intensity started at 45% (upper limbs) and 50% (lower limbs) of maximal stimulator output, increasing in 5% increments until MEP amplitude reached 1 mV or no further increase was observed. Trials affected by artefacts or poor quality were excluded. MEPs were recorded across multiple excitation levels, producing a series of EPTS values. Following clinical expert recommendations, only the EPTS corresponding to the maximal PPA was retained for analysis as the most representative and reliable for predictive modelling.

As model input, we incorporated the key motor EPTS features identified by Yperman et al. (37). Specifically, the original study employed a time series feature extraction by using highly comparative time series analysis (HCTSA) (54), followed by a multi-step feature selection pipeline that included mutual information-based filtering, hierarchical clustering to remove redundant features, and the Boruta algorithm (55) to identify the most relevant predictors for MS disability worsening. The most relevant feature 201 for the APB muscle (i.e., *EPTS Sliding Window Feature*) was found to characterize how quickly the time series returns to an average baseline following an initial peak. For the AH muscle, an autoregressive modelling-based feature (i.e., *EPTS Mean Absolute Lag-1 Autocorrelation of Prediction Errors*) demonstrated strong predictive value. For this feature, a high value means the EP still carries a clear, repeating pattern that the simple autoregressive model could not remove, whereas a low value means that, after accounting for the obvious trends, the signal exhibits a behavior similar to a random background noise. For comparability with prior literature, we also incorporated established electrophysiological biomarkers such as latency and PPA.

### 2.4 Definition of disability worsening

The prediction outcome of disability worsening was defined following established criteria (56) based on changes in the EDSS between two different time points: the baseline measurement t0, corresponding to the MRI acquisition, and the closest EDSS evaluation two to three years later t2y. Worsening was defined using the thresholds outlined in Equation 1. Consequently, each participant was assigned a binary outcome: worsened or stable (non-worsened). A single follow-up EDSS evaluation at t2y was considered sufficient for determining disability worsening, rather than requiring a subsequent confirmatory assessment. This choice increased the number of usable training instances.

(1) $$w=\{\begin{array}{ll} 1 & \text{if }{\text{EDSS}}_{t_{2y}}-{\text{EDSS}}_{t_{0}}\geq 1.5\;\&\;{\text{EDSS}}_{t_{0}}=0\;\\ 1 & \text{if }{\text{EDSS}}_{t_{2y}}-{\text{EDSS}}_{t_{0}}\geq 1\;\&\;0<{\text{EDSS}}_{t_{0}}\leq 5.5\;\\ 1 & \text{if }{\text{EDSS}}_{t_{2y}}-{\text{EDSS}}_{t_{0}}\geq 0.5\;\&\;{\text{EDSS}}_{t_{0}}>5.5\;\\ 0 & \text{otherwise}. \end{array}$$

### 2.5 Final cohort description

**Figure 1:**
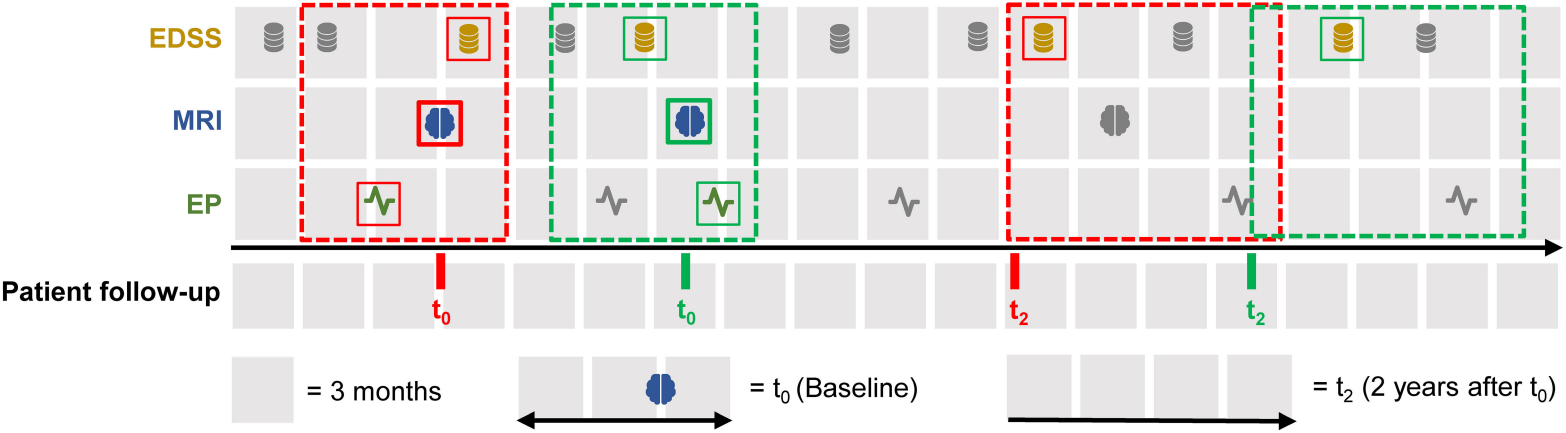
Inclusion criteria illustrated for a single patient’s follow-up timeline. This figure shows how one patient may contribute multiple episodes based on the inclusion criteria. Each episode is defined by the presence of a baseline MRI at time point *t*_0_ (blue icon), EDSS (orange icon) and EP (green icon) assessments within 6 months before to 3 months after *t*_0_, and a follow-up EDSS between 2 to 3 years later (*t*_2_). If multiple EDSS or EP measurements occur in the baseline window, the one closest to the MRI is selected. If several EDSS assessments are available beyond two years, the one closest to the 2-year mark is used for *t*_2_. Red and green dashed boxes indicate two different episodes fulfilling the inclusion criteria for one patient. Grey icons represent assessments that fall outside of the inclusion criteria. EDSS, Expanded disability status scale, EP, Evoked potentials, MRI, Magnetic resonance imaging.

Patients were included in the final cohort if they had: (1) an MRI scan at baseline (*t*_0_), (2) a motor EP and EDSS assessment performed within six months before to three months after *t*_0_, and (3) an EDSS evaluation between two and three years after the baseline (*t*_2_*_y_*). If multiple EDSS or EP assessments were available within the baseline window, the measurement closest to the MRI date was selected. Likewise, when several EDSS assessments were available beyond two years, the one closest to the two-year mark was used for $t_{2y}$. A complete case analysis was performed, excluding episodes with missing data on any required modality. **Figure 1** illustrates the inclusion criteria for the final cohort. A two-year prediction horizon was selected following the approach of De Brouwer et al. (20).

**Figure 2:**
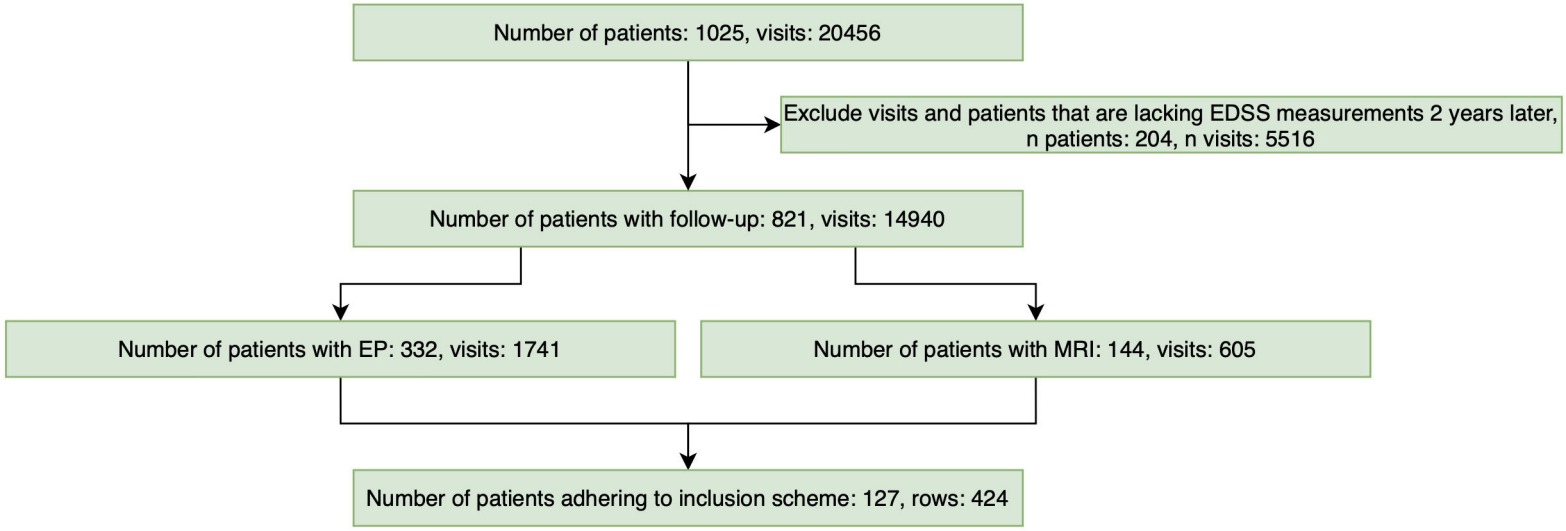
Cohort selection based on the inclusion criteria. Data inclusion started from the initial iMed export of 1,025 patients and 20,456 clinical visits. Records lacking EDSS follow-up at least two years later were excluded. Patients with available motor EP and MRI data were then filtered based on the inclusion criteria: a baseline MRI, matched EDSS and EP assessments around *t*_0_, and a follow-up EDSS at $t_{2y}$. The final dataset comprised 127 patients contributing 424 episodes. EDSS, Expanded disability status scale; EP, Evoked potentials; MRI, magnetic resonance imaging.

The clinical data export from iMed (iMed, ^©^2022 MSBase Foundation, Australia) initially included 1,025 unique patients and 20,456 clinical visits. Following the exclusion of visits lacking an EDSS follow-up at least two years later, 821 patients with a total of 14,940 visits remained. Within this subset, 332 patients had available EPTS data (1,741 visits), and 144 patients had corresponding MRI data (605 visits). As some individuals had multiple assessments meeting the inclusion criteria, their longitudinal records contributed several, occasionally overlapping, clinical episodes. After applying the full inclusion criteria, the final cohort comprised 127 unique patients contributing 424 eligible episodes. The distribution of the number of episodes contributed per patient is shown in **Appendix 9**. All statistical and ML analyses were conducted using the episode-level data. A flowchart detailing the inclusion process is shown in **Figure 2**.

In summary, for the predictive modelling, the input consisted of baseline clinical data (EDSS, age, and gender), MRI-derived anatomical and radiomics features, and motor EPTS features, while a binary outcome indicated whether disability worsening had occurred two to three years after the baseline.

At the patient level, individuals in the final cohort had a mean age of 42.3 years (± 11.9 SD) and a mean EDSS score of 2.5 (± 1.6 SD) at baseline (*t*_0_). The cohort was predominantly female (75.6%), with the majority diagnosed with relapsing-remitting MS (RRMS, *n* = 105), followed by secondary-progressive MS (SPMS, *n* = 17), primary-progressive MS (PPMS, *n* = 4), and clinically isolated syndrome (CIS, *n* = 3). A total of 16 patients (12.6%) were classified as worsened, meaning they experienced EDSS-based disability worsening in at least one episode during follow-up. The cohort represented a heavily treated population, with 76.4% receiving moderate to high DMTs. All patients met the McDonald criteria applicable at the time of diagnosis (57). Summary statistics at the episode level are presented in **Table 1**. Overall, 5.2% of the total episodes showed EDSS worsening. The remaining values reflect the underlying patient-level characteristics.

**Table 1:** Descriptive statistics of all recorded clinical episodes in the final dataset.

MS type	n	Mean age (SD)	Mean EDSS (SD)	%Females	%EDSS worsening
Total	424	41.6 (11.0)	2.2 (1.3)	75.2	5.2
RRMS	365	40.4 (10.5)	1.9 (1.0)	75.3	3.0
SPMS	47	47.4 (8.9)	4.2 (1.4)	77.0	19.1
PPMS	7	63.5 (8.7)	5.1 (1.5)	71.4	14.3
CIS	5	44.5 (14.4)	1.7 (0.4)	60.0	20.0

*n* refers to the number of recorded clinical episodes (rows) included in the final dataset. One patient may have contributed multiple episodes. CIS, Clinically isolated syndrome, EDSS, Expanded disability status scale, PPMS, Primary-progressive MS, RRMS, Relapsing-remitting MS, SD, Standard deviation, SPMS, Secondary-progressive MS.

### 2.6 Statistical analysis

For the statistical analysis, two-tailed Mann-Whitney U-tests were used to assess differences in all features between the worsening and stable patients prior to feature selection. This non-parametric test was selected because it does not assume normality of the data. To account for multiple comparisons, the Benjamini–Hochberg procedure was applied to control the false discovery rate, and the Bonferroni correction was used to reduce the risk of type I errors. Statistical significance was defined as *p< α* = 0.05. All analyses were performed using Python 3.10.12, pandas 2.2.2 (58) and NumPy 1.26.4 (59). Statistical analyses were carried out using pingouin 0.5.4 (60) and SciPy 1.14.0 (61).

### 2.7 Machine learning analysis

**Figure 3:**
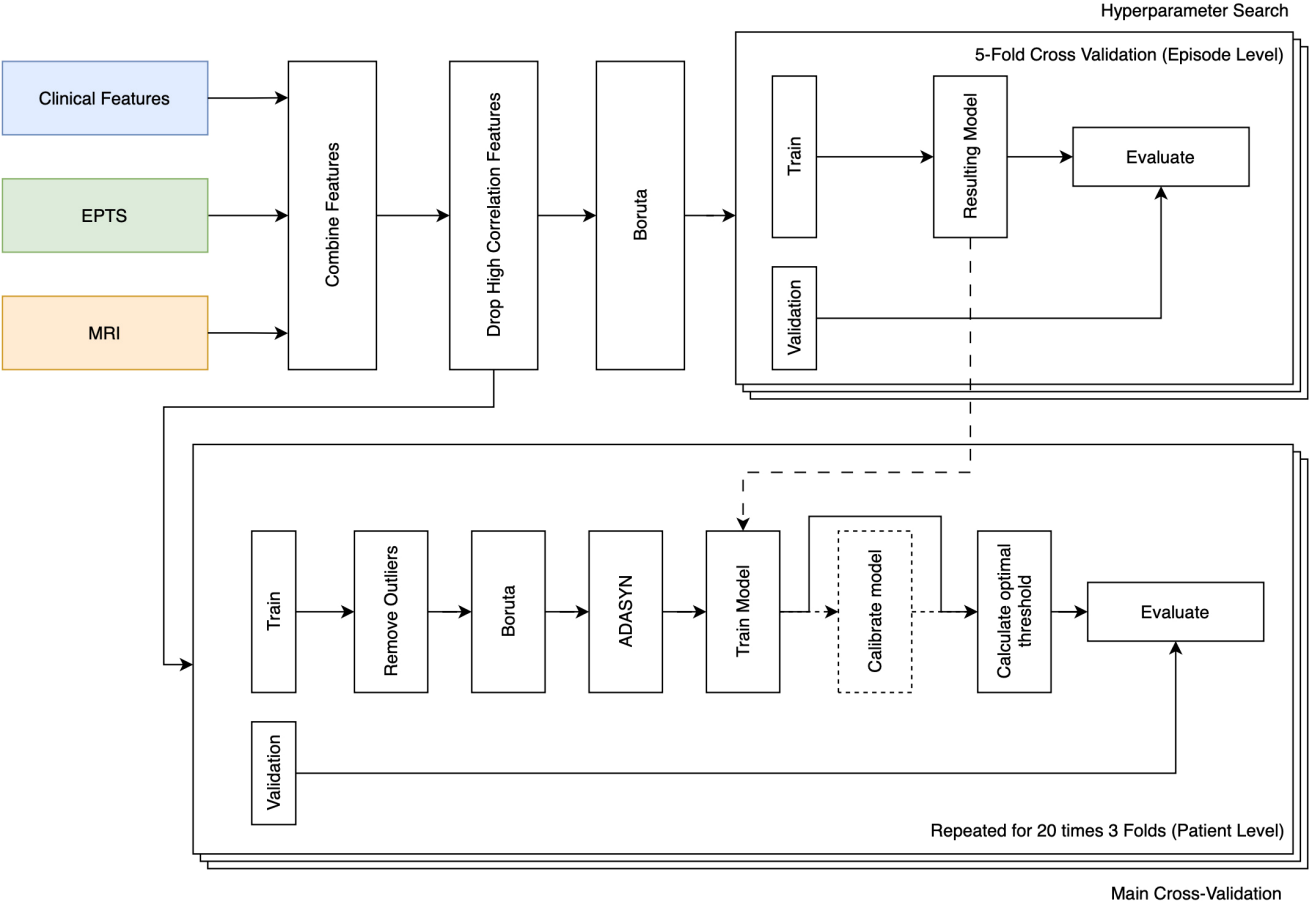
ML pipeline for predicting disability worsening in MS based on clinical, EPTS, and MRI features. The pipeline consists of three main stages. The first stage (top left) comprises the general feature extraction, where the clinical (blue), EPTS (green), and MRI radiomics (orange) features are combined according to the inclusion criteria. Next, highly correlated features (Pearson |*r*| *>* 0.9) are removed, retaining only one representative from each correlated group. The Boruta algorithm is then applied to retain a representative number of features for the next stage. The second stage (top right) was the initial hyperparameter search to determine appropriate hyperparameters for the given model setup. The resulting hyperparameters are used as the base hyperparameters for stage three. The third stage (bottom) represents the full cross-validation pipeline. This scheme includes outlier removal, Boruta, ADASYN, model training using previously optimized hyperparameters, calibration using Platt scaling, and optimal threshold determination. Model performance is evaluated within each fold of the cross-validation. ADASYN, Adaptive synthetic sampling; EPTS, Evoked potential time series; MRI, Magnetic resonance imaging.

To assess the viability and potential benefits of integrating motor EPTS and MRI features, an extensive ML analysis was conducted. The objective was to evaluate the predictive performance of both modalities, MRI radiomics and motor EPTS, separately, as well as in combination, with or without the inclusion of clinical variables. The entire ML pipeline is illustrated in **Figure 3**. A total of 96 different model setups were explored, systematically varying five key parameters:

1. Combination of modalities: EPTS, MRI and the combination of both (EPTS and MRI) are tested.
2. Choice of model: The analysis considered a range of ML models, namely Logistic Regression (LR), RF (36), Balanced RF (BRF) (62), and LGBM (25).
3. Motor EPTS feature representation: The motor EPTS features were either aggregated into a single summary statistic or retained as separate input variables to assess whether preserving the full feature space improved predictive performance.
4. Incorporation of clinical data: We evaluated all models and modality combinations with and without including the clinical variables, more specifically, the baseline EDSS, gender, and age.
5. Calibration: Models were tested with and without *post-hoc* calibration using Platt Scaling (63).

A key aspect of our methodology was the use of a repeated stratified patient 3-fold cross-validation scheme, which ensures that each patient’s data is exclusively used in either training or testing, thereby minimizing overfitting. Given the low prevalence of disability worsening in the dataset (22 worsening episodes; 5.2%), a single fixed hold-out test split would have yielded only a very limited number of positive class instances in the test set, resulting in unstable and high-variance performance estimates. Accordingly, three folds were constructed with approximately equal numbers of worsening episodes (stratified), while assigning all episodes from a given patient to a single fold to prevent within-patient information leakage. We then repeat this process 20 times, analogous to the framework proposed by Yperman et al. (37). This ensures there are enough worsening and stable patients in each test set to obtain valid metrics. The repetitions ensure that different divisions of the dataset are explored. In the following sections, we provide a detailed breakdown of our ML framework *within* this cross-validation scheme. A visual overview is shown in **Figure 3**.

#### 2.7.1 Investigated models

The ML models selected for comparison in this study included LR, RF (36), BRF (62), and LGBM (25). These models were chosen to represent a range of classification paradigms, from simple linear models (LR) to ensemble-based decision tree methods (RF and BRF) and advanced boosting techniques (LGBM). LR and RF are widely used in ML classification tasks. LR is a linear model that performs well with small datasets and serves as a strong baseline. RF, in contrast, is an ensemble method that constructs multiple decision trees, improving classification performance in complex cases, though it can be prone to overfitting. BRF is a variation of RF designed to address class imbalance. Unlike standard RF, which performs random sampling with replacement, BRF applies a balanced bootstrapped sampling strategy, ensuring equal representation of classes in each bootstrap sample. Finally, LGBM was selected as a state-of-the-art classifier for structured (tabular) data. LGBM is based on gradient boosting (64), an advanced ML technique that builds multiple decision trees sequentially, with each tree correcting the errors of the previous ones. All ML models were implemented using scikit-learn 1.5.1, with the exception of LGBM (25), which utilized its own scikit-learn-compatible library (lightgbm 4.5.0).

#### 2.7.2 Removal of highly correlated features

After applying the selection criteria detailed in 2.5, the resulting dataframe contained many highly correlated features. Features that have an absolute Pearson correlation coefficient higher than 0.9 with another feature were considered highly collinear and removed, retaining only one representative feature from each correlated pair. This is done to reduce the dimensionality of the problem and prevent overfitting on highly collinear features. The resulting dataframe is used for both the initial hyperparameter search, and subsequently the main cross-validation loop.

#### 2.7.3 Initial hyperparameter search

First, a hyperparameter search was conducted for each model and modality configuration (i.e., MRI alone, EPTS alone, and their combination with or without clinical data). To achieve this, a feature selection procedure (Boruta (55)) was applied. We retained all EPTS and clinical features throughout the selection process, as their effectiveness has been demonstrated in a previous study (37). Following feature selection, a hyperparameter optimization process was conducted using a stratified 5-fold cross-validation. The AP metric was used to evaluate the model’s performance during this search, explained in **Appendix 4**. This metric provides a single-number summary of classification performance across different thresholds, making it particularly useful for imbalanced datasets. The specific hyperparameter configurations explored are detailed in **Appendix 5**. Notably, for LR, hyperparameters were tuned using a grid search, whereas for the other models, a random search approach was employed. For each setup, optimal hyperparameters were identified. The resulting model for that specific setup was used in the final cross-validation scheme to assess predictive performance.

#### 2.7.4 Feature selection, model training & post-processing

Feature selection and preprocessing followed a structured, fold-specific strategy, as outlined below. Also, here, we retained all EPTS and clinical features throughout the selection process.

1. Outlier removal: Extreme values in the training set were removed using a *z*-score threshold of 6, meaning any observation of more than six SDs from the mean was excluded. This prevents rare but highly atypical data points from influencing the model.
2. Scaling: All features were standardized by adjusting their mean to 0 and their variation (or SD) to 1. This ensures that features with larger numerical ranges do not disproportionately affect the model’s learning process.
3. Feature selection using Boruta: We applied the Boruta algorithm (55) to determine which input features were most relevant for predicting disability worsening. This method systematically evaluates the importance of each feature by comparing it to permutations of the feature values, known as *‘shadow features’*. If a feature does not consistently outperform these permutations, it is considered uninformative and removed. To ensure that potentially useful predictors were not prematurely excluded, we applied a relatively liberal selection criterion, retaining features that outperformed their shadow counterparts in at least 75% of the comparisons.
4. Balancing class distributions with adaptive synthetic sampling (ADASYN): Because fewer patients experienced disability worsening than remained stable, the classes were imbalanced. To mitigate this imbalance, we employed the ADASYN method (65) to create synthetic examples of the minority group (i.e., those who worsened) within the training set. This helps prevent the model from becoming biased toward predicting stability and improves its ability to detect true worsening cases.
5. Model training: The ML model was trained using the aforementioned hyperparameters optimized in preliminary evaluations to enhance predictive accuracy.
6. Probability calibration using Platt scaling (63): ML models often output raw scores rather than well-calibrated probabilities. To improve interpretability, we applied Platt scaling, which fits a logistic function to these scores to produce more reliable probability estimates. If calibration is used in a given configuration, a three-fold stratified cross-validation is used to train the model 3 times. The predictions on their test sets are used to get unbiased predictions. These are then used to obtain the weights for the aforementioned Platt scaling.
7. Threshold selection: Finally, we determined the best cut-off value for classifying a patient as ‘stable’ vs. ‘worsening’. This threshold was chosen to maximize balanced accuracy, ensuring similar predictive performance for both groups rather than favoring the more common outcome. An inner validation split is made from the training data, containing 15% of the data. This is used for unbiased probability estimates, on which the optimal cut-off is determined.
8. Evaluation: The aforementioned steps result in a trained model, which is evaluated on the test set of the current fold. To this end, the following metrics are computed: Balanced accuracy, F1-Score, AUROC, average precision (AP), sensitivity, specificity, and Brier score.

#### 2.7.5 Evaluation of all scenarios with *Post-Hoc* testing and critical difference diagram

Following model training within the cross-validation scheme, we evaluated performance using multiple metrics computed for each fold. Given the severe class imbalance in our dataset, we reported both the AUROC and AP. While AUROC is a widely used benchmark, it can overestimate model performance in imbalanced datasets, as it considers both true positive and true negative classifications equally. AP, by contrast, provides a more stringent evaluation by focusing on the model’s ability to correctly identify cases of disability worsening (the minority class). To further assess predictive reliability, we included the Brier score, which quantifies the accuracy of predicted probabilities by measuring the mean squared difference between predicted and actual outcomes. A complete overview of all evaluation metrics is provided in **Appendix 4**.

As previous studies in MS have focused on different evaluation metrics, often either AUROC or AP, it is informative to examine the trade-off between these metrics. This can be achieved by plotting the performances of each model on a two-dimensional scatter plot. To further interpret model performance, the Pareto front can be visualized, consisting of the configurations of models that dominate other configurations in a bi-objective setup (66). For example, when evaluating AUROC and AP, each point on the Pareto front corresponds to a Pareto-optimal model configuration that achieves the highest possible AUROC for a given AP or the highest AP for a given AUROC.

To determine the most effective combination of data modalities, a Friedman test was performed. Model performance was assessed using the three aforementioned evaluation metrics: AUROC, AP, and Brier score. These metrics are chosen as they are not sensitive to a threshold, and more accurately reflect the models’ performance in an unbalanced setting (AUROC and AP), with the models’ probabilistic performance in mind (Brier score). The other reported metrics (Balanced accuracy, F1-score, sensitivity, and specificity) are dependent on the choice of threshold on the output probabilities of the models. Each metric was normalized by subtracting its mean and dividing by its SD. First, for each cross-validation repetition, the normalized metrics were averaged, yielding 20 values per model setup. These were then averaged to obtain an overall performance estimate. To identify the best-performing modality combination for each repetition, the maximum average score was selected across all models. The resulting values were used to construct a critical difference diagram, following the methodology described by Demšar (67).

When the Friedman test yielded a statistically significant result, a *post-hoc* Wilcoxon signed-rank test (68) was conducted to compare the relative rankings of the best models per modality configuration and identify significant differences between those. The resulting rankings and critical difference diagrams facilitated the identification of the most informative and robust modality combinations for predicting MS disability worsening.

#### 2.7.6 Interpretability

To enhance model interpretability, SHapley Additive exPlanations (SHAP) (69) were used. SHAP values quantify the contribution of each feature to individual model predictions, capturing both the magnitude and direction of their influence. Summary plots were generated to visualize overall feature importance and to indicate whether specific features were associated with an increased or decreased likelihood of disability worsening.

## 3 Results

### 3.1 Statistical significance of features

**Figure 4:**
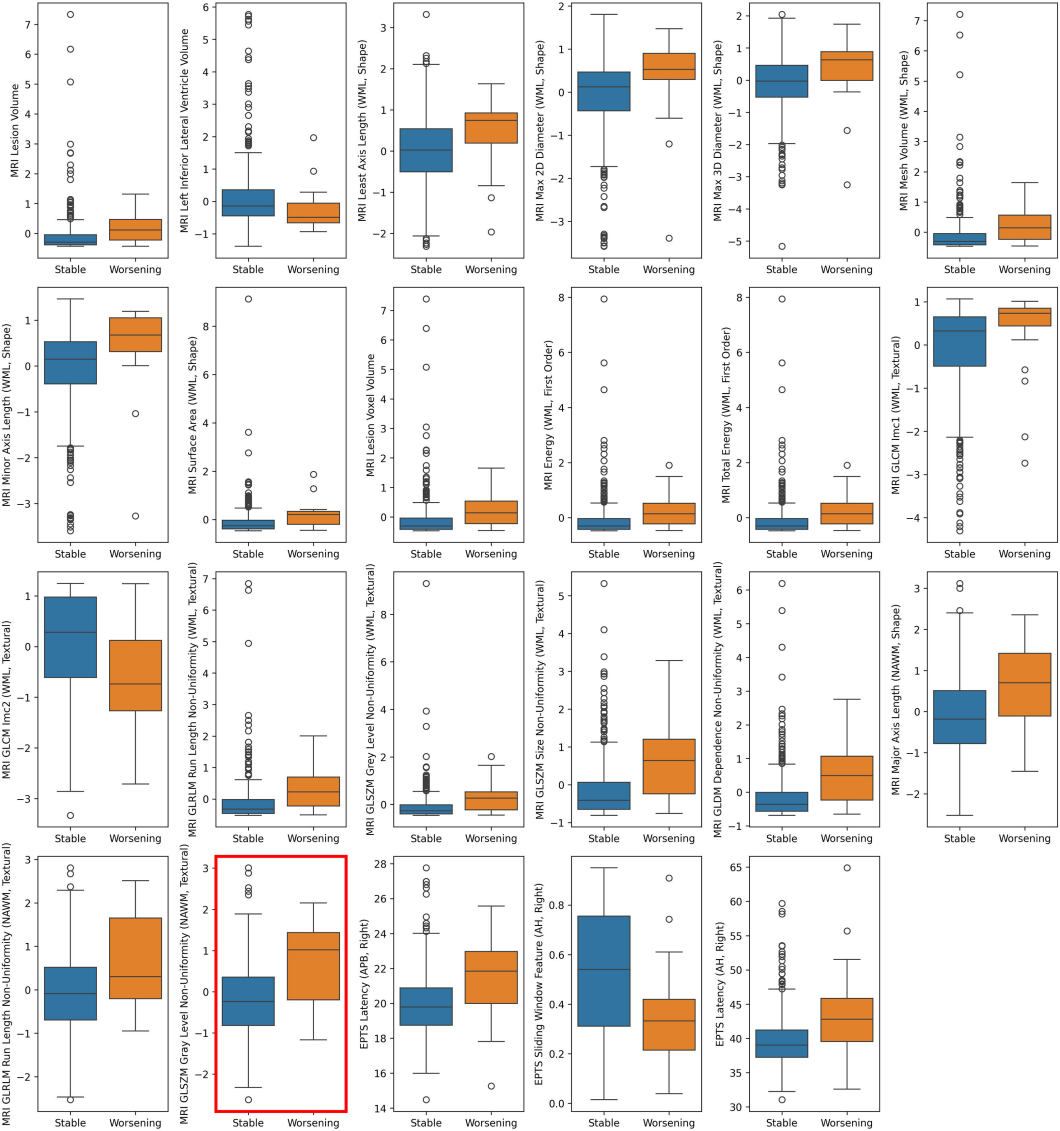
Features that differed significantly between stable and worsening patients. Paired boxplots displaying the distribution of all features that remained statistically significant after the Mann-Whitney U test with Benjamini-Hochberg false discovery rate correction. Each boxplot compares feature distributions between patients with disability worsening (*n* = 22) and those who remained stable (*n* = 402). The median, interquartile range, and outliers are shown for each feature. This analysis identified 23 significant features, suggesting that multiple clinical or imaging-derived measures differ between the two patient groups when using a less conservative correction method. *GLSZM Grey Level Non-Uniformity* of the NAWM was the only feature differing between worsening and stable patients after Bonferroni correction (*p* = 0.035), marked with a red outline. AH, Abductor halluces; APB, Abductor pollicis brevis; EP, Evoked potentials; EPTS, Evoked potential time series; GLCM, Grey Level Co-occurrence Matrix; GLDM, Grey Level Dependence Matrix; GLRLM, Grey Level Run Length Matrix; GLSZM, Grey Level Size Zone Matrix; Imc, Informational Measure of Correlation; MRI, Magnetic resonance imaging; NAWM, Normal-appearing white matter; WML, White matter lesions; PPA, Peak-to-peak amplitude.

To identify features that differed significantly between stable and worsening groups on an episode level, we performed a Mann-Whitney U test, applying Bonferroni correction. After correction, only one feature remained statistically significant, specifically *GLSZM Grey Level Non-Uniformity of the NAWM* (*p*-value = 0.035), as shown with a red outline in **Figure 4**. This feature quantifies signal heterogeneity in the NAWM, with higher values observed in patients who experienced disability worsening. If, instead, the less strict Benjamini-Hochberg correction was used, 23 features were identified as significant. Boxplots for these 23 features are also displayed in **Figure 4**. These included 8 textural radiomics features (e.g., *GLRM Run Length Non-Uniformity of the WML*), 10 shape-based or anatomical features (e.g., *Lesion Volume, Surface Area of the WML*), 2 First-order statistics features (e.g., *Total Energy of the WML*), and three EPTS-derived features (e.g., *EPTS Latency (APB, Right)*). Most of these features showed significantly higher median values in the worsening group.

### 3.2 Comparison of model performance across metrics

**Figure 5:**
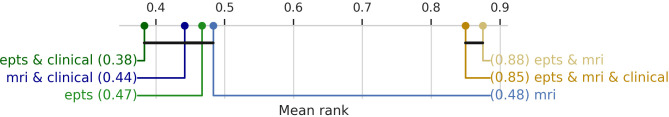
Critical difference diagram comparing the performance of different data modality combinations across all experiments and evaluation metrics. The critical difference diagram illustrates the average rank of each data modality configuration across all folds based on model performance for every evaluated metric, including AUROC, AP, and Brier score. A higher rank indicates superior overall performance. Statistical significance was assessed using a Friedman test (*p* = 1.9 · 10^−9^), followed by a *post-hoc* Wilcoxon signed-rank test with Holm correction to determine pairwise differences between modality configurations. Modality configurations that are connected by a horizontal line do not exhibit statistically significant differences, while those that are separated belong to significantly different performance groups. The results suggest that models integrating both EPTS and MRI data achieve superior rankings compared to single-modality models. AUROC, Area under the receiver operating characteristic curve; AP, Average precision; EPTS, Evoked potential time series; MRI, Magnetic resonance imaging.

To statistically assess whether the combination of different data modalities resulted in a significant performance advantage over multiple metrics, we employed a critical difference diagram (**Figure 5**). Statistical significance was first determined using a Friedman test (*p* = 1.9 · 10^−9^), followed by a *post-hoc* Wilcoxon test with Holm correction to assess pairwise differences between models (67). The figure shows that the combined feature sets perform better with statistical significance, compared to the modalities alone. No significance can be determined when clinical features are present together with the combined feature set, nor are there any significant differences between the modalities alone.

**Figure 6:**
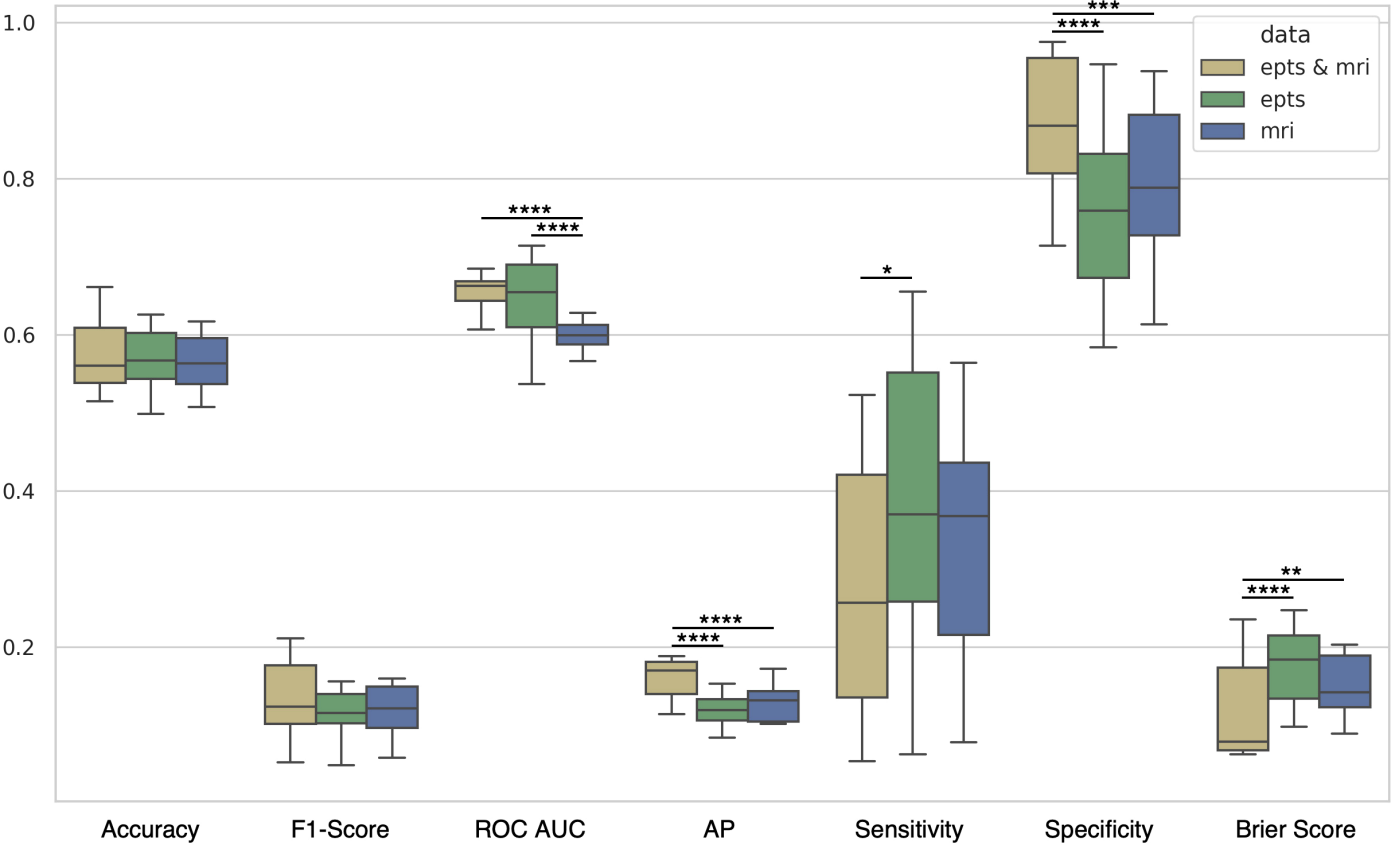
Comparison of model performance across model configurations. Boxplots displaying the distribution of key performance metrics across all cross-validation folds for each model configuration. The evaluated metrics include balanced accuracy, F1-score, AUROC, AP, sensitivity, specificity, and Brier score. Statistical significance is denoted with asterisks (∗*p<* 0.05, ∗∗*p<* 0.01, ∗∗∗*p<* 0.001, ∗∗∗∗*p<* 0.0001). Each modality represents the data with the inclusion and exclusion of clinical variables. Models integrating both EPTS and MRI data exhibit slight improvements in median performance across multiple metrics. AP, Average precision; AUROC, Area under the receiver operating characteristic curve; EPTS, Evoked potential time series; MRI, Magnetic resonance imaging.

**Figure 6** presents a comparative analysis of model performance across various evaluation metrics (detailed in **Appendix 4**). The boxplots illustrate the distribution of performance scores across cross-validation folds, with slight improvements observed in models incorporating both motor EPTS and MRI data. Each modality and combination plotted represents the data with the inclusion and exclusion of clinical data, as no significant differences were observed in **Figure 5**. These models exhibited statistically significant superior median performance compared to the other modalities alone in AP, specificity, and Brier score. Additionally, the combination performs better with statistical significance than MRI radiomics features alone when considering AUROC.

### 3.3 Metric trade-offs

#### 3.3.1 AUROC and AP

**Figure 7:**
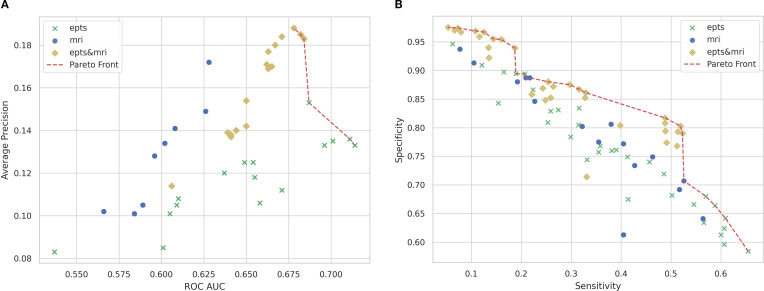
Performance trade-offs for predicting disability worsening across modality combinations. The figure presents two key performance comparisons across different model configurations: **(a)** The relationship between mean AUROC and mean AP over all folds for each tested model configuration. The Pareto front, highlighted in red, consisted of only EPTS and combined EPTS-MRI models, with EPTS models leading in AUROC and combined models achieving the highest AP. **(b)** The trade-off between mean sensitivity and mean specificity over all folds for each tested model configuration, where models incorporating both EPTS and MRI features achieved a better balance. EPTS-only models exhibited the highest sensitivity, suggesting stronger detection of patients likely to experience disability worsening. AP, Average precision; AUROC, Area under the receiver operating characteristic curve; EPTS, Evoked potential time series; MRI, Magnetic resonance imaging.

**Figure 7a** illustrates the trade-off between two key performance metrics: AUROC and AP. The results indicate that models based on MRI features consistently achieved higher AP scores, whereas EPTS-based models had superior AUROC performance. Notably, the Pareto front, representing models that optimize one metric without sacrificing the other, comprised only EPTS and combined EPTS-MRI models. Among these, EPTS-only models outperformed all others in AUROC, whereas models integrating EPTS and MRI features achieved the highest AP, suggesting superior predictive performance in identifying patients at risk of worsening.

#### 3.3.2 Sensitivity and specificity

The trade-off between sensitivity and specificity is depicted in **Figure 7b**. Models incorporating both EPTS and MRI data demonstrated overall superior performance, achieving a better balance between sensitivity (correctly identifying patients with worsening disability) and specificity (correctly identifying stable patients). Notably, EPTS-only models exhibited the highest sensitivity, indicating a stronger ability to detect patients who would experience worsening disability.

### 3.4 Feature importance and interpretability of model with lowest brier score

**Figure 8:**
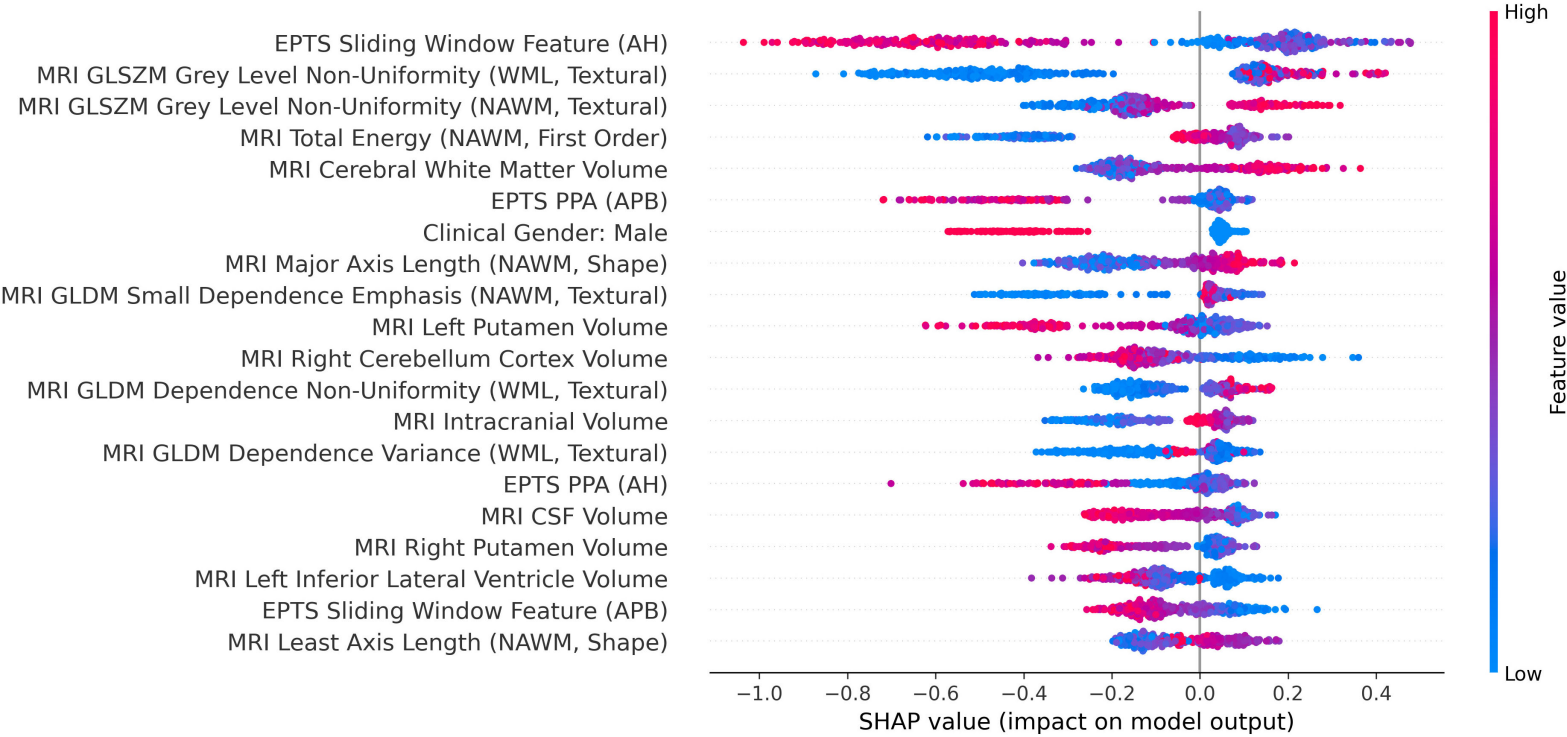
Feature importance and SHAP values for the best-performing model based on Brier score. Each feature is ranked by its overall importance in the model’s predictions, with the most influential features at the top. Each point represents a single prediction instance, with the SHAP value on the x-axis indicating the feature’s impact on the model output. The color scale represents feature values: blue indicates lower values and red indicates higher values. Higher SHAP values suggest a stronger contribution toward predicting disability worsening. AH, Abductor Hallucis; APB, Abductor pollicis brevis; CSF, Cerebrospinal fluid; EPT, Evoked potential time series; GLDM, Grey Level Dependence Matrix; GLSZM, Grey Level Size Zone Matrix; MRI, Magnetic resonance imaging; NAWM, Normalappearing white matter; PPA, Peak-to-peak amplitude; SHAP, Shapley additive explanations; WML, White matter lesions.

Since there are many trade-offs concerning the metrics reported by the different models, the Brier score is chosen to determine the best-performing model. The model with the lowest Brier score (0.062) is an LGBM model (hyperparameters in **Appendix 7**). **Figure 8** presents the SHAP summary plot. **Appendix 8** contains the numerical SHAP values corresponding to the features in the plot. The majority of high-impact features in the model were derived from MRI radiomics. These included textural features such as *GLSZM Grey Level Non Uniformity*, which reflects heterogeneity within NAWM and lesion areas. Higher values were associated with an increased likelihood of disability worsening, suggesting that greater heterogeneity in brain tissue is linked to disease progression. Shape-based MRI features also played a significant role, particularly those related to brain atrophy, such as the *Major and Least Axis Length* of the NAWM. In these cases, lower values were associated with a higher probability of worsening, indicating that reduced structural integrity in non-lesional tissue contributes to the model’s predictions.

In addition to MRI-based features, several EPTS features emerged as important predictors. Notably, *Sliding Window*–based measures ranked highly in the SHAP analysis, with increased values generally corresponding to a greater risk of disability worsening. Furthermore, the PPA of EPTS signals was selected as a relevant feature, where lower values were linked to a higher probability of disability worsening.

Among clinical features, gender was the only variable retained by the model. SHAP values indicated that male gender was associated with a lower predicted risk of disability worsening compared to female gender.

## 4 Discussion

In this study, we introduced and evaluated a multimodal ML pipeline that integrates clinical data, MRI radiomics, and motor EPTS to predict long-term disability worsening in PwMS. By combining structural changes observed in MRI with subclinical functional alterations revealed by EPTS, our primary aim was to enhance prognostication beyond single-modality models, thereby improving the early identification of individuals at increased risk for worsening disability. This early detection could empower clinicians to make more informed and timely treatment decisions, ultimately reducing long-term neurological damage.

### 4.1 Multimodal outperforms single modality

In our cross-validation framework, all configurations and metrics evaluated demonstrated that combining high-dimensional MRI and EPTS features outperformed models that relied solely on one modality. This finding confirms the additional value of multimodal approaches. Structural and functional biomarkers from distinct data modalities, imaging and time series, appear to provide complementary information about disease processes that may not be captured by any single assessment. Moreover, we utilized high dimensional features instead of conventional measures, revealing subtle structural changes in seemingly normal tissue and functional disturbances that might otherwise go undetected (24, 37).

### 4.2 MRI feature insights

Consistent with previous research on brain texture analysis in MS (70–73), texture-based features of WMLs, most prominently *GLSZM Gray Level Non-Uniformity* and *GLDM Dependence Non-Uniformity*, were found to be associated with subsequent worsening of disability (74). The former feature quantifies the dispersion of signal intensities within a lesion, while the latter captures the variability in the spatial grouping of similar intensities. Higher values for either feature indicate greater heterogeneity and structural irregularity.

Similarly, increased textural complexity in the NAWM, exemplified by *GLSZM Grey Level Non*-*Uniformity*, also predicted EDSS worsening in both the statistical and SHAP interpretability analyses (73, 74). Together, these findings support the idea that diffuse microstructural abnormalities in both WML and NAWM reflect underlying processes such as demyelination, inflammation, and axonal loss (75, 76). In line with this, longitudinal quantitative MRI data have shown that reductions in myelin and axon volume fractions in the NAWM precede the development of more destructive lesions, such as T1-weighted hypointense black holes, further highlighting the prognostic relevance of early microstructural disruption (77).

Shape-based features of the NAWM, specifically the *Least Axis Length* and *Major Axis Length*, also appeared in SHAP analyses. These features measure the smallest and largest axes, respectively, of a three-dimensional ellipsoid that encompasses the ROI in the brain (78). Higher values in these shape measures may indicate swelling or more extensive structural alterations in the NAWM. This suggests that atrophy or morphological distortions in non-lesional tissue can have significant prognostic implications (79).

Furthermore, certain anatomical features, such as atrophy in the cerebellum or putamen, have been identified as predictors of worsening disability, supporting the established correlation between damage to subcortical and cerebellar structures and declines in motor and cognitive functions (80–83). In contrast, other anatomical features related to atrophy, such as the volumes of white matter, cerebrospinal fluid, and lateral ventricles, did not effectively distinguish between patients with stable disease and those experiencing disability worsening, which is inconsistent with earlier research (84). This inconsistency may arise from patients exhibiting significant atrophy being categorized as stable due to their already elevated baseline EDSS scores. Consequently, the two-year follow-up period may not have been long enough to capture meaningful EDSS worsening in this subgroup (see **Appendix 3**). Additionally, factors like variability in imaging between centers, reliance on reconstructed FLAIR MRI, and class imbalance may also contribute to the differences observed between our findings and previous studies.

### 4.3 EPTS feature insights

Most of the features selected by our ML model were derived from MRI data, likely due to the larger number of radiomics-based inputs. However, certain EPTS features emerged among the top-ranking selections. Notably, the *Sliding Window*-type features, which were previously identified as highly relevant from a comprehensive pool of 7,700 motor EPTS features by Yperman et al. (37), were confirmed again in our study. Yperman et al. explain that this feature is calculated by moving a window equal to half the length of the EPTS across the signal in 25% increments, resulting in three overlapping segments. For each window, the mean is computed, and the SD of these means is divided by the SD of the full EPTS. Essentially, this metric quantifies the variability of the temporal signal after the initial stimulation peak, providing an indirect measure of how quickly and consistently the signal returns to baseline. While its precise physiological interpretation remains unclear, the repeated selection of this feature across different modalities suggests that it carries meaningful prognostic information. This insight may be particularly relevant in the context of a well-treated and clinically stable cohort, where traditional clinical and imaging markers might lack sensitivity to detect subtle progression. In such cases, the prognostic value of EPTS features could lie in their ability to reflect subclinical activity that is otherwise undetectable. Further research is needed to explore the underlying physiological significance of this feature.

In addition to the *Sliding Window*-type features, our model also identified *PPA* in the APB muscle as a key predictor, which contrasts with the findings from Yperman et al. (37), where incorporating amplitude-based features did not consistently enhance model performance. One possible explanation for this discrepancy is that, in our multimodal dataset, PPA gained importance through synergistic interactions with a wide range of MRI radiomics features, thereby increasing its overall predictive contribution.

### 4.4 Clinical features insights

In our SHAP analysis, gender emerged as the only relevant clinical feature. Notably, being male was associated with a lower predicted risk of worsening disability. This finding contradicts the common belief that men typically experience worse outcomes in MS (85, 86). One possible explanation for this result is that men constituted a much smaller percentage of our cohort, reflecting the typical female-to-male ratio seen in MS (87, 88). Also, fewer men experienced EDSS worsening or exhibited MRI or EPTS abnormalities detected by our model (6 men compared to 16 women). As a result, the model may have learned that male gender was less correlated with the subtle functional and structural deficits associated with MS progression. Another contributing factor may be the use of synthetic oversampling. Given the small number of worsening episodes, ADASYN-generated samples may outnumber real positive cases, increasing the risk that the models might be biased. Moreover, the observed effect may reflect center-specific clinical decision-making, whereby male patients might have received more intensive treatment based on the well-known prognostic factors, potentially leading to a lower observed rate of progression in this group. Thus, the apparent protective effect of being male in our analysis could be due to cohort-specific biases rather than a genuine difference in disease progression between sexes.

### 4.5 Clinical implications

While the exact physiological mechanisms underlying high-dimensional motor EPTS features are not yet fully understood, our findings demonstrate that these functional measures carry substantial prognostic information. EPTS features consistently ranked among the most informative predictors, even when evaluated alongside a large set of MRI radiomics features, indicating that electrophysiological markers capture disease-related information that is distinct from, and complementary to, structural imaging. This complementarity is particularly relevant in the context of progression independent of relapse and MRI activity (PIR(M)A) (89–91), where patients may continue to accumulate disability despite stable conventional MRI and absence of relapses, posing the increasingly important clinical question of how to identify such patients at an earlier stage.

Interestingly, while the model combining MRI and EPTS data generally achieved the best overall balance across most performance metrics, EPTS-only models demonstrated the highest AUROC and sensitivity. This suggests that EPTS alone may be more effective at identifying those few individuals who are likely to experience disability worsening, even in an imbalanced dataset where predicting the minority class was essential. High sensitivity is particularly important in a prognostic context, as it helps minimize the chances of giving false reassurance to patients who are actually at risk. Since we did not analyze the temporal sequence of changes across modalities in this study, future longitudinal research should investigate whether alterations in EPTS occur before structural imaging abnormalities. At the same time, MRI radiomics features remained essential in our best-performing models, underscoring the established role of MRI imaging in the diagnosis and monitoring of MS.

From a clinical perspective, incorporating MEP-based measures into routine evaluations for MS could be beneficial, given their relatively low cost and general feasibility. Brief MEP sessions impose a minimal additional burden and may uncover functional impairments in PwMS who appear clinically stable or radiologically unchanged, making them suitable for interim monitoring of patients between scheduled MRI examinations. In this context, multimodal prediction frameworks could, once externally validated, be applied during routine follow-up to flag patients at increased risk of disability worsening despite apparent clinical and MRI stability. Such risk stratification could motivate closer monitoring, adapted follow-up intensity, or additional functional assessments, and may support earlier consideration of treatment adaptation, particularly in light of emerging therapeutic strategies targeting progression. Early identification of individuals at risk for worsening disability, therefore, has the potential to inform both monitoring strategies and treatment decision-making in a manner aligned with a patient’s prognostic profile (9, 92).

Finally, the integration of MRI radiomics and EPTS highlights the utility of ML approaches for combining high-dimensional structural and functional data. Such multimodal quantitative patterns, which arise from complex mathematical transformations of the input data, are difficult to detect solely through visual inspection or conventional summary measures. ML methods can therefore augment clinical interpretation by capturing non-linear relationships and interactions that are not readily apparent to the clinician’s eye. At the same time, the features contributing to these predictions should be viewed as mathematical descriptors of complex patterns rather than as directly interpretable physiological markers. Consequently, the present work represents a proof of concept, and our findings provide quantitative support for multimodal follow-up strategies in MS and motivate future validation of multimodal, high-dimensional ML approaches in larger, multicenter cohorts.

### 4.6 Limitations and future directions

This study has several limitations that must be considered. First, the small sample size (127 patients) and low proportion of disability progression events (5.2%) limited statistical power and introduced substantial class imbalance, and within-patient dependencies. This phenomenon reflects the proactive treatment environment of a tertiary MS center, where fewer patients accumulate measurable disability. To counter this limitation, we employed a repeated stratified patient K-fold cross-validation, ensuring that episodes from one patient never appeared in both training and test sets. While this method introduces some bias due to its repetitive nature, it effectively addresses the inherent variance of our small cohort. Additionally, ADASYN oversampling was applied to synthesize additional minority-class examples, enhancing our analysis.

Although these steps partially mitigate overfitting and imbalance-related bias, we acknowledge that they do not fully compensate for the small sample size and skewed distribution of outcomes. The large whiskers observed in our boxplots reflect this variability across validation folds and highlight the fragility of performance estimates in a limited dataset. Future work should aim to validate these findings using larger, multicenter datasets, thereby enhancing statistical power and the robustness of performance estimates. Nevertheless, we evaluated model performance using a variety of metrics, including both discrimination (e.g., AUROC, AP) and calibration measures (e.g., Brier score), to examine the trade-off between competing objectives. Across nearly all of these metrics and their combinations, the multimodal framework encompassing both MRI radiomics and EPTS features, with or without clinical variables, consistently outperformed single-modality models.

Another limitation is the exclusion of DMT use as a predictive feature, which may have introduced confounding, as different treatment regimens significantly impact progression risk. This omission was due to incomplete or heterogeneous treatment data in this retrospective cohort. Nevertheless, the high proportion of patients receiving moderate-to-high efficacy therapies reflects contemporary clinical practice and underscores that measurable disability worsening can still occur despite effective treatment. Future models should incorporate detailed longitudinal treatment data, such as DMT type, timing, and duration, to enhance clinical relevance. Moreover, our data-driven approach may not sufficiently account for existing biases in the dataset, such as the notable gender imbalance inherent to MS prevalence, which could distort model outputs.

Third, disability worsening was defined using a binary outcome based on a single EDSS assessment approximately two years after the baseline assessment. Confirmed disability progression could not be reliably assessed in the present retrospective cohort due to limited visit density. As a result, this outcome definition may capture transient EDSS fluctuations and does not fully exploit the granularity of the continuous EDSS scale, and may be less sensitive in patients with higher baseline EDSS scores. Nevertheless, this pragmatic choice was made to ensure a sufficient number of events and comparability with prior real-world prognostic studies. Future work should incorporate denser longitudinal follow-up to enable confirmed progression endpoints over a longer period and continuous modelling of EDSS trajectories to avoid bias in outcome ascertainment.

Fourth, conducting a hyperparameter search prior to the cross-validation could introduce slight bias, as hyperparameters may have been exposed to testing sets, potentially inflating observed performance metrics. However, because this process was uniformly applied across all configurations, the comparative power of the study (EPTS vs. MRI and their combination) remains intact.

Fifth, the use of multiple MRI acquisition protocols and different EP devices for data collection inevitably introduces heterogeneity in feature extraction. We sought to mitigate this through MRI harmonization and through stringent standardization of EP acquisition and processing at the center of data collection, thereby minimizing variability across devices. Nevertheless, the presence of multiple MRI acquisition protocols necessitated the use of a SRR approach using PRETTIER (45), as radiomics analysis requires three-dimensional MRI data (24). While our results indicate that radiomics features derived from the NAWM contributed more strongly to the predictive models, this should not be interpreted as evidence that lesion-based features are of lesser biological relevance. Rather, it is plausible that bias introduced during image reconstruction and subsequent automated lesion segmentation affected lesion representation, thereby reducing the apparent importance of lesion radiomics features.

Sixth, an important limitation of this study is the absence of external validation. All data were acquired within a single center and a single country, using a Philips 1.5T scanner with three distinct acquisition protocols. Independent external validation in larger, multicenter cohorts is required to confirm the robustness and transportability of the results. Although MRI reconstruction using SRR was applied to reduce technical variability, the generalizability of the proposed models to other centers, scanners, and patient populations cannot be assessed. Accordingly, our findings should be interpreted as proof-of-concept evidence supporting the potential value of multimodal data integration rather than as a clinically deployable prediction tool. We further note that harmonization approaches similar to those applied here have been successfully used in multicenter neuroimaging studies and may facilitate future external validation across scanners and sites (24). However, such validation is beyond the scope of the present dataset.

Lastly, the complex preprocessing required for obtaining both MRI radiomics and EPTS features poses challenges for generalizability and limits the clinical applicability of our findings. The limited interpretability of high-dimensional features, alongside their derivation at a group level, presents obstacles to effectively translating model outputs into individual clinical decisions. Nonetheless, these features have the potential to capture subtle structural and functional abnormalities that clinicians often perceive during examinations but may struggle to quantify objectively.

## 5 Conclusion

Overall, it is evident from this study that combining MRI radiomics with motor EPTS features yields a beneficial effect on predictive performance for MS disability worsening, outperforming single-modality models across multiple performance metrics. As the first effort to combine these modalities, our findings provide strong proof-of-concept for multimodal ML-based prognostication in MS. Validation in larger, multicenter cohorts is essential to confirm these results and enable clinical translation. Future studies should also explore additional modalities, longitudinal methods, and deep learning approaches to further enhance personalized risk stratification in MS.

## Data Availability

The datasets presented in this article are not readily available because this is confidential routine clinical data and hence authorization for making it public have not been granted. Requests to access the datasets should be directed to liesbet.peeters@uhasselt.be.

## Footnotes

1 www.statistical-modelling.de/lst.html.

2 http://www.fil.ion.ucl.ac.uk/spm.
