# Supplementary figures and images for: Combining magnetic resonance imaging and evoked potentials enhances machine learning prediction of multiple sclerosis disability worsening
Source: Front Immunol. 2026 Mar 11;17:1625837. doi: 10.3389/fimmu.2026.1625837 (PMC13013075; doi:10.3389/fimmu.2026.1625837)

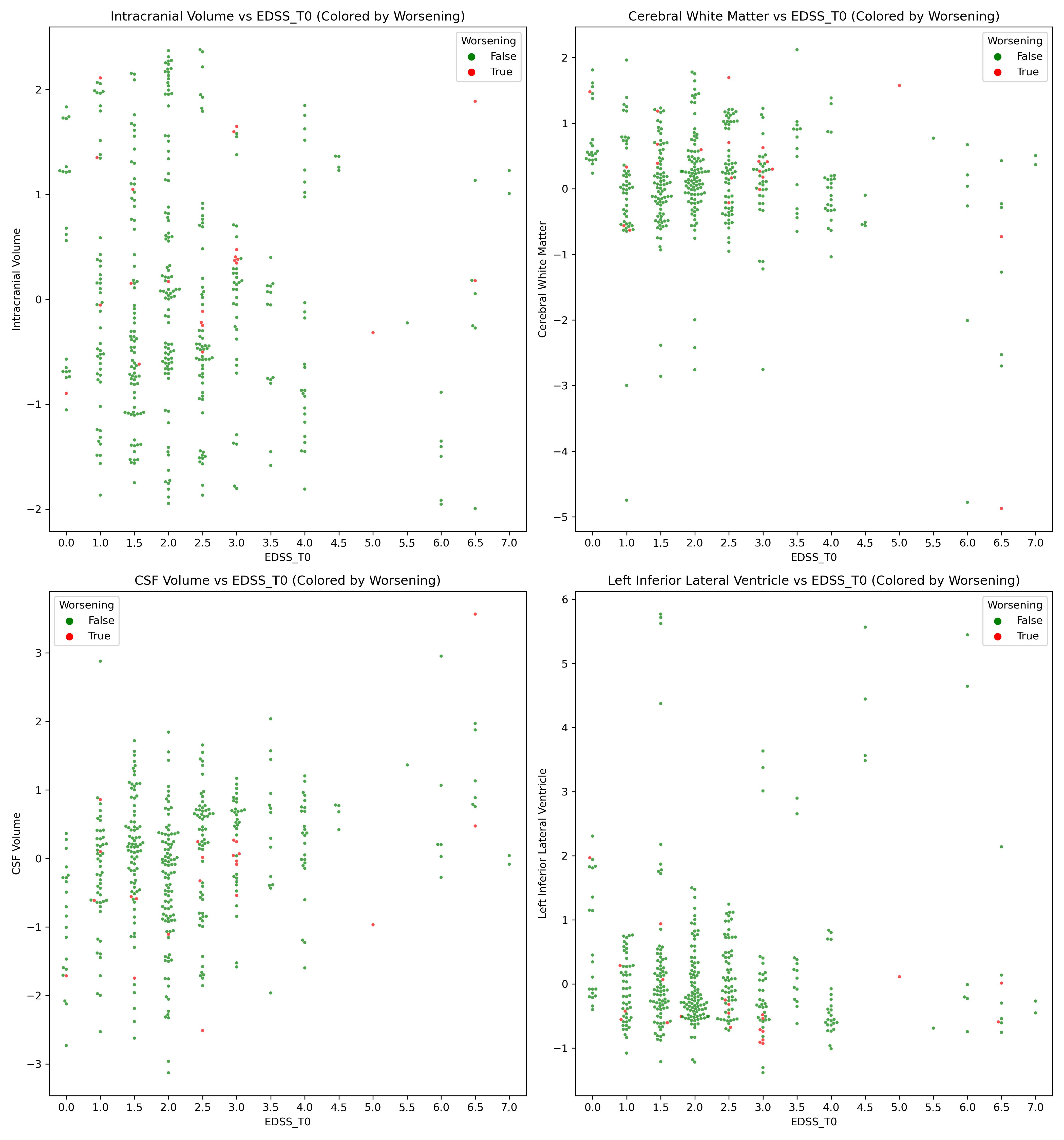

Supplement: Supplementary file 1 [file Image1.jpeg]

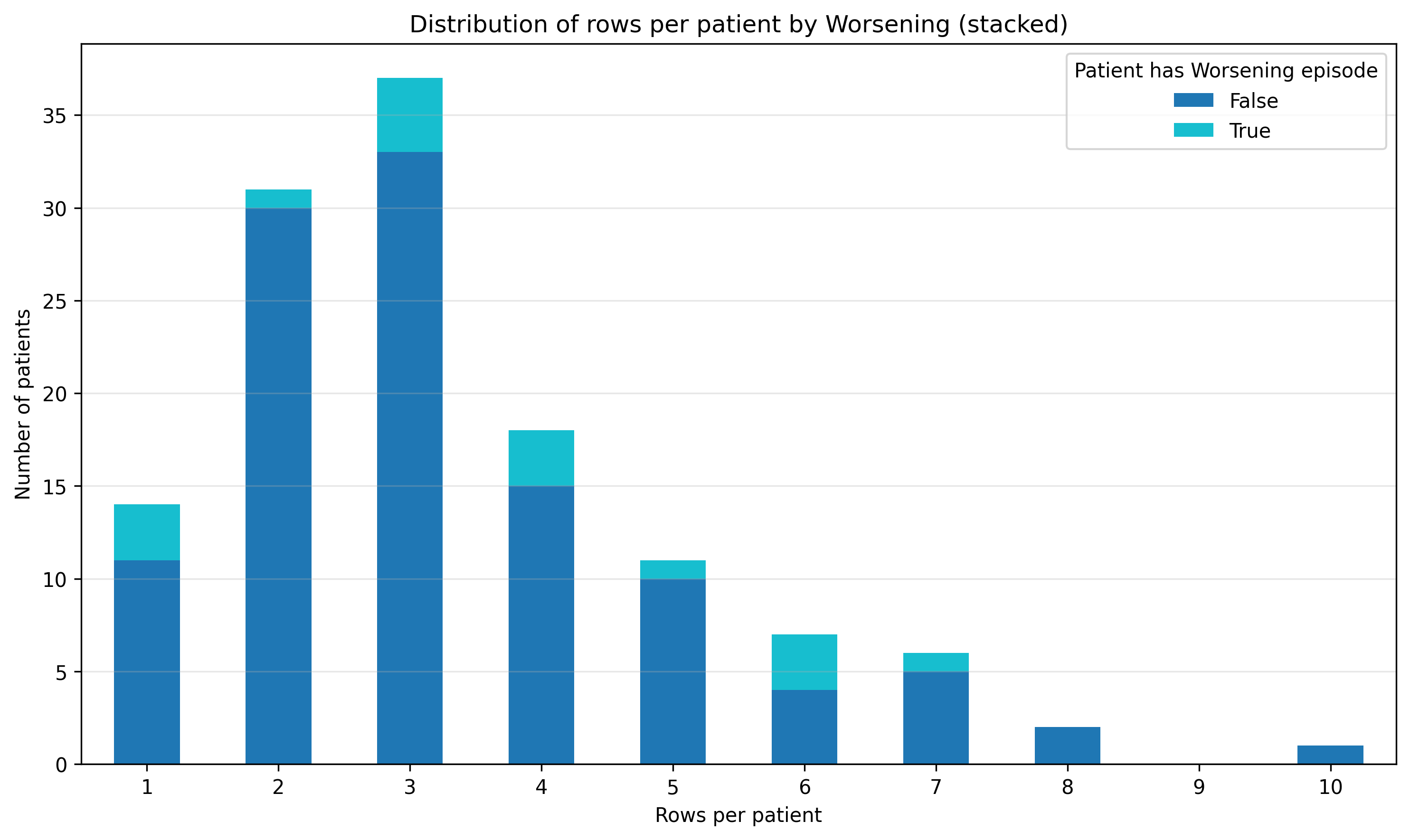

Supplement: Supplementary file 2 [file Image2.png]
