# Supplementary material for: Combining magnetic resonance imaging and evoked potentials enhances machine learning prediction of multiple sclerosis disability worsening
Source: Front Immunol. 2026 Mar 11;17:1625837. doi: 10.3389/fimmu.2026.1625837 (PMC13013075; doi:10.3389/fimmu.2026.1625837)
Supplement: Supplementary file 4 [file DataSheet2.pdf]

APPENDIX 1 MRI ACQUISITION PARAMETERS

Table 1.1. General acquisition details.

General details of the acquisitions				
**Protocol**	**# Sessions**	**Scanner**	**Acquisition Type**	**Images per Session**
A	328	Philips Achieva 1.5T	2D	2
B	44	Philips Achieva 1.5T	3D	3
C/CsT1	53	Philips Achieva 1.5T	3D	1

Table 1.2. Timing parameters.

Timing Parameters			
**Protocol**	**Repetition Time (ms)**	**Echo Time (ms)**	**Inversion Time (ms)**
A	6000	120	2000
B	4800	321 - 363	1660
C/CsT1	4800	323 - 356	1660

Table 1.3. Spatial parameters.

Spatial Parameters			
**Protocol**	**Slice Thickness (mm)**	**Slice Spacing (mm)**	**Pixel Spacing (mm)**
A	5.0	6.0	0.72 - 0.90
B	3.0	3.0	0.98
C/CsT1	1.2	0.6	0.98

APPENDIX 2 NUMBER OF RADIOMICS FEATURES PER CLASS

Table 2.1. Summary of features extracted per Region of Interest (ROI).

Feature Class	#Features Extracted per ROI
Shape	14
First-order statistics (FO)	18
Grey Level co-occurrence matrix (GLCM)	22
Grey Level run length matrix (GLRLM)	16
Grey Level size zone matrix (GLSZM)	16
Grey Level dependence matrix (GLDM)	14

APPENDIX 3 RELATIONSHIP BETWEEN BASELINE EDSS AND BRAIN VOLUME MEASURES, COLOURED BY DISABILITY WORSENING

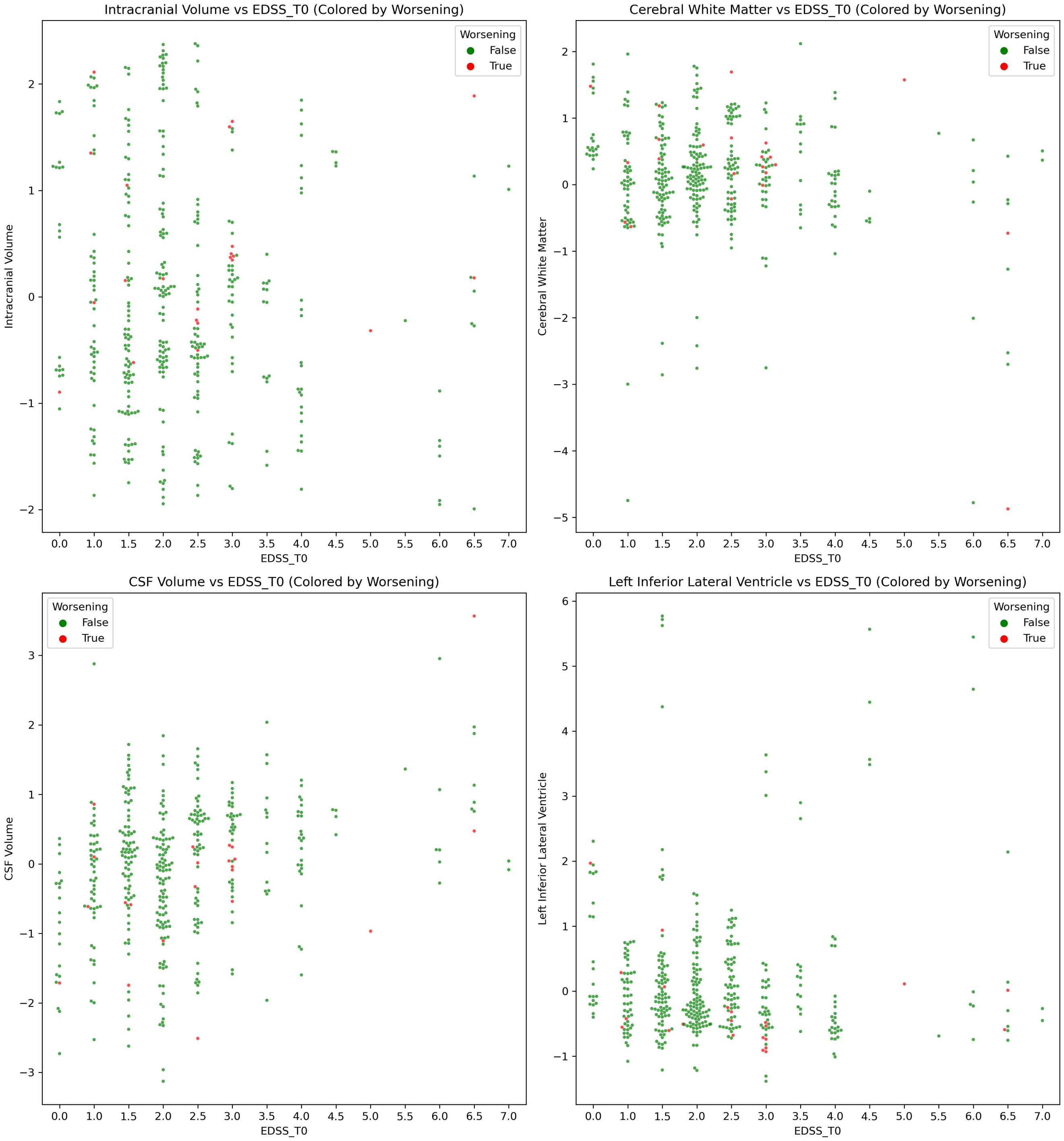

Figure 3.1. Scatter plots showing the relationship between baseline EDSS (EDSS_t0) and various brain volume measures, with data points colour-coded based on disability worsening status (green = stable, red = worsening). The plots illustrate: (Top-left) Intracranial volume vs. EDSS_T0, (Top-right) Cerebral white matter volume vs. EDSS_T0, (Bottom-left) CSF volume vs. EDSS_T0, and (Bottom-right) Left inferior lateral ventricle volume vs. EDSS_T0. The distribution highlights potential associations between brain atrophy and disability progression in multiple sclerosis.

APPENDIX 4 PERFORMANCE METRICS

Various performance metrics are used in this study. The following paragraphs explain the different metrics and their importance in imbalanced classification.

Appendix 4.1 Balanced Accuracy, Sensitivity & Specificity

Balanced Accuracy is a metric used to evaluate the performance of a classification model, especially in imbalanced datasets. It is the average of sensitivity and specificity, providing a balanced view of the model's performance on both classes. The formula for Balanced Accuracy is:

$$\text{Balanced Accuracy} = \frac{\text{Sensitivity} + \text{Specificity}}{2}$$

where Sensitivity (True Positive Rate) and Specificity (True Negative Rate) are defined as:

$$\text{Sensitivity} = \frac{TP}{TP + FN}, \quad \text{Specificity} = \frac{TN}{TN + FP}$$

Here, TP is True Positives, TN is True Negatives, FP is False Positives, and FN is False Negatives.

Appendix 4.2 F1-Score

The F1-Score is the harmonic mean of precision and recall (sensitivity), providing a single metric that balances both concerns. It is particularly useful for imbalanced datasets. The F1-Score is calculated as:

$$\text{F1-Score} = 2 \cdot \frac{\text{Precision} \cdot \text{Recall}}{\text{Precision} + \text{Recall}}$$

where Precision and Recall are defined as:

$$\text{Precision} = \frac{TP}{TP + FP}, \quad \text{Recall} = \frac{TP}{TP + FN}$$

Appendix 4.3 AUROC

The Area Under the Receiver Operating Characteristic curve (AUROC) measures the model's ability to distinguish between classes. It is the area under the ROC curve, which plots the True Positive Rate (Sensitivity/Recall) against the False Positive Rate (1 - Specificity) at various thresholds. The AUROC ranges from 0 to 1, where 1 indicates perfect classification. The formula for the ROC curve is:

$$\text{ROC Curve} = \{(\text{FPR}(t), \text{TPR}(t)) \mid t \in \text{thresholds}\}$$

where $\text{FPR}(t) = \frac{FP}{FP+TN}$ and $\text{TPR}(t) = \frac{TP}{TP+FN}$.

Appendix 4.4 Average Precision

Average Precision (AP) summarizes the precision-recall curve as the weighted mean of precisions achieved at each threshold, with the increase in recall from the previous threshold used as the weight. It is

useful for imbalanced datasets. The formula for AP is:

$$\text{AP} = \sum_n (R_n - R_{n-1}) P_n$$

where P_n and R_n are the precision and recall at the n-th threshold.

Appendix 4.5 Brier Score

The Brier Score measures the accuracy of probabilistic predictions. It is the mean squared difference between the predicted probabilities and the actual outcomes. The Brier Score ranges from 0 to 1, where 0 indicates perfect accuracy. The formula is:

$$\text{Brier Score} = \frac{1}{N} \sum_{i=1}^{N} (p_i - y_i)^2$$

where p_i is the predicted probability, y_i is the actual outcome (0 or 1), and N is the number of predictions.

APPENDIX 5 HYPERPARAMETER SEARCH PARAMETERS

Table 5.1. Hyperparameter tuning grid for Logistic Regression

**Hyperparameter**	**Search Space**
Regularization Strength (C)	$\log_{10} C \in [10^{-4}, 10^{1}]$, 16 values
L1 Ratio (ℓ_1)	$\ell_1 \in [0, 1]$, 15 values

Table 5.2. Hyperparameter tuning grid for Random Forest

**Hyperparameter**	**Search Space**
Number of Trees (n)	$n \sim$ Uniform$(10, 1000)$
Max Depth (d)	$d \sim$ Uniform$(1, 10)$
Max Features	$\{\sqrt{\text{features}}, \log_2(\text{features})\}$
Class Weight	{balanced, balanced_subsample}
Min Samples Split (s)	$s \sim$ Uniform$(2, 10)$
Min Samples per Leaf (l)	$l \sim$ Uniform$(1, 10)$
Bootstrap	{True, False}

Table 5.3. Hyperparameter tuning grid for Balanced Random Forest

**Hyperparameter**	**Search Space**
Number of Trees (n)	$n \sim \text{Uniform}(10, 1000)$
Max Depth (d)	$d \sim \text{Uniform}(1, 10)$
Max Features	$\{\sqrt{\text{features}}, \log_2(\text{features})\}$
Min Samples Split (s)	$s \sim \text{Uniform}(2, 10)$
Min Samples per Leaf (l)	$l \sim \text{Uniform}(1, 10)$
Bootstrap	$\{$True, False$\}$

Table 5.4. Hyperparameter tuning grid for LGBM

**Hyperparameter**	**Search Space**
Number of Trees (n)	$n \sim \text{Uniform}(10, 1000)$
Learning Rate (η)	$\eta \sim \text{LogUniform}(10^{-4}, 10^{0})$
Number of Leaves (l)	$l \sim \text{Uniform}(2, 100)$
Feature Fraction (f)	$f \sim \text{Uniform}(0.1, 1.0)$
Bagging Fraction (b)	$b \sim \text{Uniform}(0.1, 1.0)$
Max Depth (d)	$d \sim \text{Uniform}(1, 10)$
Max Bins (m)	$m \sim \text{Uniform}(2, 255)$
Min Data in Leaf (min_l)	$\text{min}_l \sim \text{Uniform}(1, 100)$
Min Sum Hessian in Leaf (h)	$h \sim \text{LogUniform}(10^{-4}, 10^{2})$

APPENDIX 6 LIST OF ANATOMICAL FEATURES

Table 6.1. Anatomical Features Included in the Analysis

1	Left cerebral cortex volume
2	Right cerebral cortex volume
3	Left cerebral white matter volume
4	Right cerebral white matter volume
5	Left cerebellum cortex volume
6	Right cerebellum cortex volume
7	Left cerebellum white matter volume
8	Right cerebellum white matter volume
9	Right amygdala volume
10	Left amygdala volume
11	Right hippocampus volume
12	Left hippocampus volume
13	Left accumbens area volume
14	Right accumbens area volume
15	Left putamen volume
16	Right putamen volume
17	Right pallidum volume
18	Left pallidum volume
19	Left caudate volume
20	Right caudate volume
21	Right thalamus volume
22	Left thalamus volume
23	Right choroid plexus volume
24	Left choroid plexus volume
25	Right ventral diencephalon volume
26	Left ventral diencephalon volume
27	Right inferior lateral ventricle volume
28	Left inferior lateral ventricle volume
29	Fourth ventricle volume
30	Third ventricle volume
31	Left lateral ventricle volume
32	Right lateral ventricle volume
33	Cerebrospinal fluid volume
34	Brain stem volume
35	Cerebral grey matter cortex volume
36	Cerebral white matter volume
37	Ventricle volume
38	Total white matter lesion volume
39	Intra cranial volume
40	Fifth ventricle volume
41	Unknowns volume

APPENDIX 7 HYPERPARAMETERS BEST LGBM MODEL

Table 7.1. Optimised Hyperparameters for LGBM Classifier with mean Brier score 0.062

Hyperparameter	Value
Bagging Fraction	0.6387
Feature Fraction	0.1855
Learning Rate	0.0032
Maximum Bin	79
Maximum Depth	9
Minimum Data in Leaf	22
Minimum Sum Hessian in Leaf	0.0044
Number of Estimators	989
Number of Leaves	86

APPENDIX 8 SHAP VALUES

Table 8.1. Feature Importance Values

Feature	SHAP Value
EPTS Sliding Window Feature (AH)	0.390698
GLSZM Grey Level Non-Uniformity (MRI Texture Feature)	0.351489
GLSZM Grey Level Non-Uniformity (NAWM)	0.177078
Total Energy (First-Order Feature, NAWM)	0.164261
Cerebral White Matter Volume	0.158671
EPTS PPA (APB)	0.142723
Gender: Male	0.141927
Major Axis Length (NAWM)	0.137957
GLDM Small Dependence Emphasis (NAWM)	0.127069
Left Putamen Volume	0.126693
Right Cerebellum Cortex Volume	0.126605
GLDM Dependence Non-Uniformity (MRI Texture Feature)	0.115301
Intracranial Volume	0.113254
GLDM Dependence Variance (MRI Texture Feature)	0.113109
EPTS PPA (AH)	0.108277
Cerebrospinal Fluid (CSF) Volume	0.106471
Right Putamen Volume	0.103338
Left Inferior Lateral Ventricle Volume	0.096826
EPTS Sliding Window Feature (APB)	0.094284
Least Axis Length (NAWM)	0.092743

APPENDIX 9 HISTOGRAM OF EPISODES

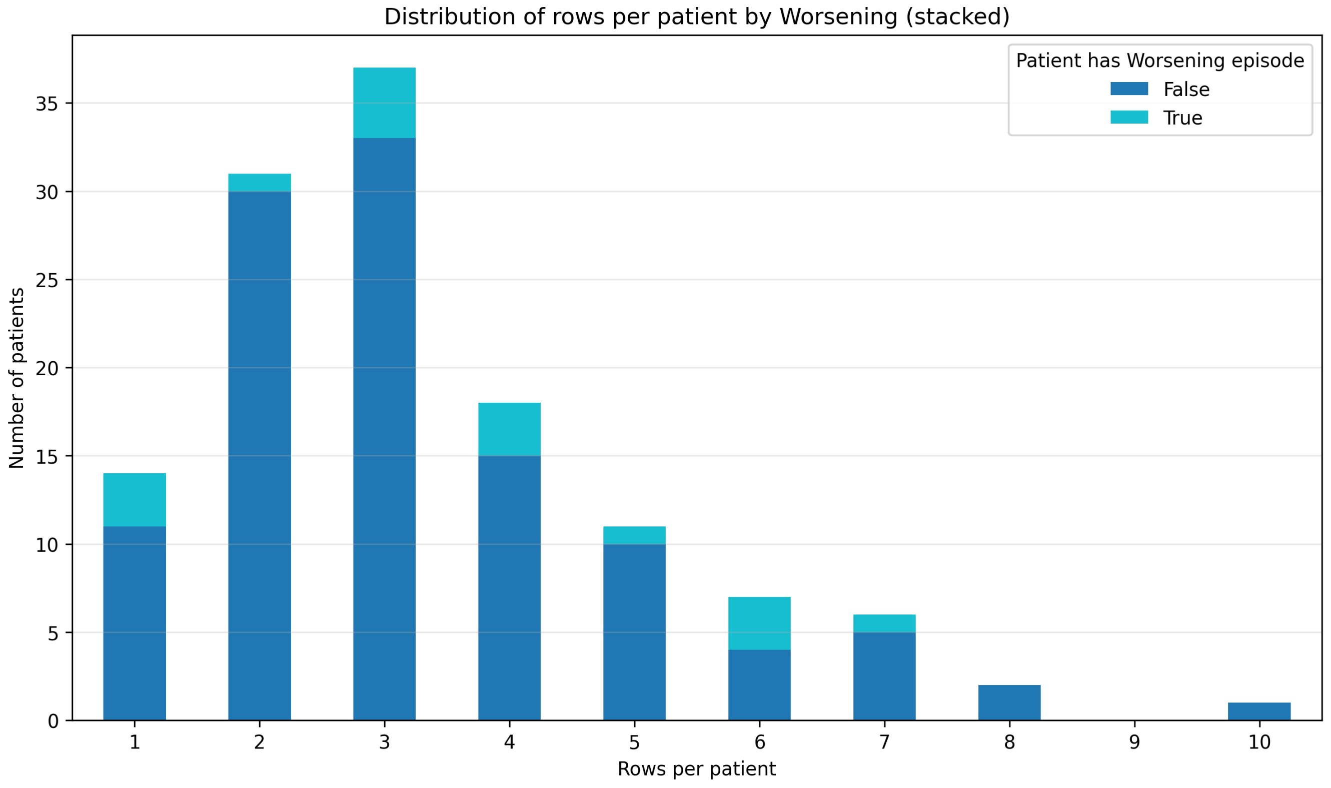

Figure 9.1. Histogram of episodes, comparing how often a certain patient appears in the dataset. This corresponds to rows in the dataset used in this study. The dark blue bars are patients who have not a single row with a worsening event, whereas the light blue bars are patients who have a row in the dataset containing a worsening label.

APPENDIX 10 TRIPOD CHECKLIST

TRIPOD

TRIPOD Checklist: Prediction Model Development

Section/Topic	Item	Checklist Item	Section
**Title and abstract**			
Title	1	Identify the study as developing and/or validating a multivariable prediction model, the target population, and the outcome to be predicted.	Title Page
Abstract	2	Provide a summary of objectives, study design, setting, participants, sample size, predictors, outcome, statistical analysis, results, and conclusions.	Abstract
**Introduction**			
Background and objectives	3a	Explain the medical context (including whether diagnostic or prognostic) and rationale for developing or validating the multivariable prediction model, including references to existing models.	1
	3b	Specify the objectives, including whether the study describes the development or validation of the model or both.	1
**Methods**			
Source of data	4a	Describe the study design or source of data (e.g., randomized trial, cohort, or registry data), separately for the development and validation data sets, if applicable.	2.1
	4b	Specify the key study dates, including start of accrual; end of accrual; and, if applicable, end of follow-up.	2.1
Participants	5a	Specify key elements of the study setting (e.g., primary care, secondary care, general population) including number and location of centres.	2.1
	5b	Describe eligibility criteria for participants.	2.5
	5c	Give details of treatments received, if relevant.	2.5
Outcome	6a	Clearly define the outcome that is predicted by the prediction model, including how and when assessed.	2.4
	6b	Report any actions to blind assessment of the outcome to be predicted.	NA
Predictors	7a	Clearly define all predictors used in developing or validating the multivariable prediction model, including how and when they were measured.	2.2 - 2.3
	7b	Report any actions to blind assessment of predictors for the outcome and other predictors.	NA
Sample size	8	Explain how the study size was arrived at.	2.5
Missing data	9	Describe how missing data were handled (e.g., complete-case analysis, single imputation, multiple imputation) with details of any imputation method.	2.5
Statistical analysis methods	10a	Describe how predictors were handled in the analyses.	2.6 - 2.7
	10b	Specify type of model, all model-building procedures (including any predictor selection), and method for internal validation.	2.7
	10d	Specify all measures used to assess model performance and, if relevant, to compare multiple models.	2.7.5
Risk groups	11	Provide details on how risk groups were created, if done.	2.7.4
**Results**			
Participants	13a	Describe the flow of participants through the study, including the number of participants with and without the outcome and, if applicable, a summary of the follow-up time. A diagram may be helpful.	2.5
	13b	Describe the characteristics of the participants (basic demographics, clinical features, available predictors), including the number of participants with missing data for predictors and outcome.	2.5
Model development	14a	Specify the number of participants and outcome events in each analysis.	2.5
	14b	If done, report the unadjusted association between each candidate predictor and outcome.	3.1
Model specification	15a	Present the full prediction model to allow predictions for individuals (i.e., all regression coefficients, and model intercept or baseline survival at a given time point).	Appendix 7
	15b	Explain how to the use the prediction model.	2.1
Model performance	16	Report performance measures (with CIs) for the prediction model.	3.2
**Discussion**			
Limitations	18	Discuss any limitations of the study (such as nonrepresentative sample, few events per predictor, missing data).	4.6
Interpretation	19b	Give an overall interpretation of the results, considering objectives, limitations, and results from similar studies, and other relevant evidence.	4
Implications	20	Discuss the potential clinical use of the model and implications for future research.	4.5
**Other information**			
Supplementary information	21	Provide information about the availability of supplementary resources, such as study protocol, Web calculator, and data sets.	2.1
Funding	22	Give the source of funding and the role of the funders for the present study.	6

We recommend using the TRIPOD Checklist in conjunction with the TRIPOD Explanation and Elaboration document.
